# Heterobimetallic Uranium(V)-Alkali Metal Alkoxides: Expanding the Chemistry of f-Block Elements

**DOI:** 10.3390/molecules30112361

**Published:** 2025-05-29

**Authors:** Andreas Lichtenberg, Lidia Inderdühnen, Aida Lichtenberg, Sanjay Mathur

**Affiliations:** Institute of Inorganic and Materials Chemistry, University of Cologne, 50939 Cologne, Germany; andreas.lichtenberg@uni-koeln.de (A.L.); lidia.inderduehnen@uni-koeln.de (L.I.); aida.lichtenberg@uni-koeln.de (A.L.)

**Keywords:** actinide, uranium, f-block, uranium(V) chemistry, alkali metal, alkoxide, coordination compound, crystal structure, molecular structure, molecular precurso

## Abstract

Heterobimetallic uranium(V) alkoxides incorporating monovalent alkali metal counterions display remarkable structural versatility, dictated by the steric demands of the alkoxide ligands and the ionic radius of the alkali metal. Compounds of the general formula [UM(O*^t^*Bu)_6_] (**UM-O*^t^*Bu**-type: M = Na, K, Rb, Cs) were obtained by: (i) reacting [U(O*^t^*Bu)_5_(py)] with equimolar amounts of alkali metal silylamides in *tert*-butyl alcohol, and (ii) oxidative transformation of [UM_2_(O*^t^*Bu)_6_] (M = Na, K, Rb, Cs) upon reaction with iodine. *Trans*-alcoholysis of uranium heterobimetallic *tert*-butoxides with sterically less demanding *iso*-propyl alcohol yields oligomeric or polymeric *iso*-propoxide derivatives of the general formula [UM(O*^i^*Pr)_6_]*_n_*, where the nuclearity depends on the alkali metal (*n* = 2 for M = Li; *n* = ∞ for M = Na, K, Rb). The capacity of alkali metal ions to adopt flexible coordination geometries results in different structural types ranging from finite clusters to infinite chains, with [ULi(O*^i^*Pr)_6_]_2_ (**ULi-O*^i^*Pr-1**) found to be dimeric, whereas [UM(O*^i^*Pr)_6_]_∞_ (**UM-O*^i^*Pr-2**-type, M = Na, K) and [URb(O*^i^*Pr)_6_]_∞_ (**URb-O*^i^*Pr-3**) exhibit a polymeric architecture. These findings provide fresh insights into the structure-directing influence of alkali metals on actinide coordination chemistry and broaden the chemistry of actinide alkoxides. All compounds were unambiguously characterized in both solution and solid-state through NMR and IR spectroscopic studies, as well as single crystal X-ray diffraction analysis.

## 1. Introduction

Uranium(V), occupying a critical redox link between the stable U(IV) and U(VI) states, exhibits distinctive chemical behavior driven by its 5f^1^ electronic configuration [1]. This intermediate oxidation state displays paramagnetic properties [2], arising from unpaired 5f electrons along with a pronounced tendency to exhibit unique spin–orbit coupling effects [3]. While often transient in aqueous media due to disproportionation tendencies (2U^5+^ → U^4+^ + U^6+^) [4], U(V) can be stabilized in solid-state compounds, such as binary U_2_O_5_ [5] or ternary MUO_3_ (M = Li [6], Na [7], K [7]) oxides, as well as molecular complexes [8], where it provides a unique platform to probe 5f-electron behavior, actinide redox mechanisms, and metal–ligand bonding in both solid-state and molecular systems [9]. However, compared to the more stable oxidation states +IV and +VI, compounds of uranium in the +V state are scantily described [3,10].

**Figure 1:**
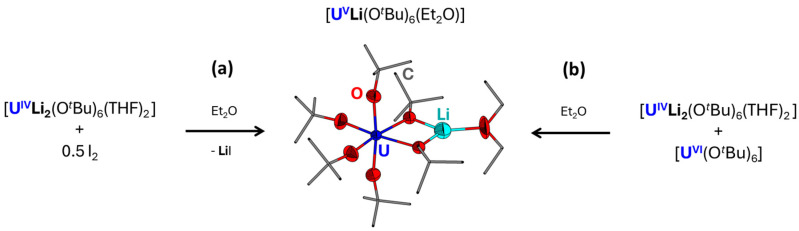
The heterobimetallic uranium(V) *tert*-butoxide [ULi(O*^t^*Bu)_6_(Et_2_O)] was synthesized via two distinct redox pathways, as reported by Hayton et al. [16]. It was successfully obtained via (**a**) oxidation of [ULi_2_(O*^t^*Bu)_6_(THF)_2_] with elemental iodine in diethyl ether, and (**b**) comproportionation of [ULi_2_(O*^t^*Bu)_6_] and [U(O*^t^*Bu)_6_] [16].

Although the synthesis of uranium(V) ethoxide and related alkoxides was first reported in the 1950s [11,12,13], only a limited number of uranium(V) alkoxides have been structurally characterized to date. Access to the pentavalent oxidation state in uranium alkoxide chemistry has been demonstrated in species such as the mononuclear [U(O*^t^*Bu)_5_(py)] [14], the dinuclear [U(O*^i^*Pr)_5_]_2_ [15], and the heterobimetallic [LiU(O*^t^*Bu)_6_(Et_2_O)] [16]. Notably, [U(O*^t^*Bu)_5_(py)] serves as a suitable starting material in the synthesis of group 14 heterobimetallic uranium(V) alkoxides, as reported by Mathur et al. [14]. The *tert*-butoxide derivative [LiU(O*^t^*Bu)_6_(Et_2_O)] was synthesized by two distinct redox pathways, as reported by Hayton et al. [16]. In the first approach, oxidation of the U(IV) alkoxide [ULi_2_(O*^t^*Bu)_6_(THF)_2_] with I_2_ (0.5 equiv.) yielded the pentavalent uranium compound [LiU(O*^t^*Bu)_6_(Et_2_O)] (Figure 1a), which was also accessible via the comproportionation reaction between [ULi_2_(O*^t^*Bu)_6_(THF)_2_] and [U(O*^t^*Bu)_6_] (Figure 1b) [16].

Heterobimetallic alkali metal alkoxides represent a versatile and significant class of coordination compounds, incorporating a broad spectrum of metals across the periodic table [17,18,19,20,21,22]. In addition to their intriguing structural chemistry, heterobimetallic alkoxides with alkali metal counterions are valuable synthetic intermediates for rational construction of heterobi- and trimetallic alkoxides, for example, via salt metathesis reactions with metal halides [17,18,19,20,23,24]. In this context, [KAl(O*^t^*Bu)_4_] serves as a prominent example, which can undergo reactions with divalent metal halides such as MgCl_2_, CoCl_2_, NiCl_2_ and CuI_2_, leading to the formation of the trinuclear spiro compound [MM’_2_(O*^t^*Bu)_8_] (M = Al; M’ = Mg, Co, Ni, Cu) [25,26,27]. Moreover, metal alkoxides act as efficient single-source precursors for the synthesis of metal oxide materials [28,29,30,31,32,33,34,35,36]. In this context, ferroelectric lithium niobate (LiNbO_3_) fibers [37] and lithium tantalate (LiTaO_3_) films [38] and powders [39] have been synthesized using [LiM(OEt)_6_] (M = Nb, Ta) as a single-source precursor, followed by calcination of the resulting decomposition product in air. In light of this, ternary alkali metal oxides are useful in energy conversion and storage systems (i.e., LiCoO_2_ [40] cathodes in LIBs and NaMnO_2_ [41] cathodes in NaIBs).

**Figure 2:**
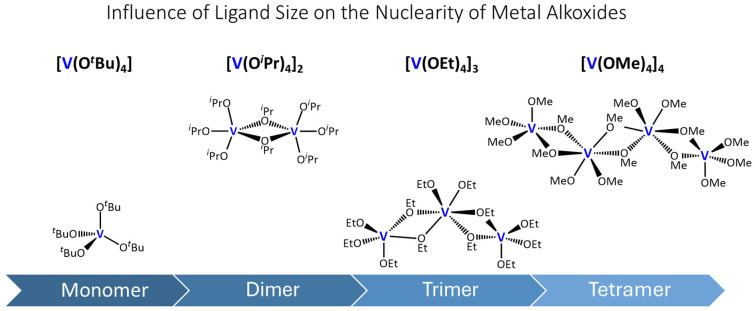
The molecular structures of vanadium(IV) alkoxides are evidently governed by the size of the alkoxide ligand. [V(OR)_4_]*_n_* exists as a monomer (*n* = 1) for the *tert*-butoxide derivative (R = *^t^*Bu), whereas the less bulky *iso*-propoxide (R = *^i^*Pr) forms a dimer (*n* = 2), ethoxide ligands (R = Et) form a trimer (*n* = 3), and methoxide ligands (R = Me) form a tetramer (*n* = 4) [44,45].

Trans-alcoholysis involving the exchange of bulkier alkoxide groups against sterically less-demanding alkoxo groups is an effective strategy to modulate the nuclearity of metal alkoxides. Reducing ligand steric bulk systematically increases oligomerization, driven by diminished steric shielding and the enhanced capacity of smaller alcoholates to form *µ*_2_ or even *µ*_3_ bridges that interconnect metal centers [42,43]. For example, homoleptic vanadium(IV) alkoxides [V(OR)_4_]*_n_* demonstrate this trend: the *tert*-butoxide derivative (R = *^t^*Bu, *tert*-butyl) adopts a monomeric structure (*n* = 1), whereas *iso*-propoxide (R = *^i^*Pr, *iso*-propyl) forms dimers (*n* = 2), ethoxide ligands (R = Et, ethyl) oligomerize as trimers (*n* = 3), and methoxide (R = Me, methyl) produces tetramers (*n* = 4) (Figure 2) [44,45].

Building on our group’s recent findings regarding the effects of alkali metal cations in heterobimetallic actinide(IV) *tert*-butoxides [46], this work extends the study to uranium(V) alkoxides to elucidate the structural dynamics across an alkali metal series (M = Li, Na, K, Rb, Cs). We report herein the synthesis and characterization of uranium(V) *tert*-butoxide complexes, [UM(O*^t^*Bu)_6_], followed by ligand exchange with smaller *iso*-propoxide ligands to assess nuclearity and connectivity changes.

## 2. Results and Discussion

### 2.1. U(V)M(I) tert-Butoxide Compounds [UM(O^t^Bu)_6_] (UM-O^t^Bu)

**Scheme 1:**
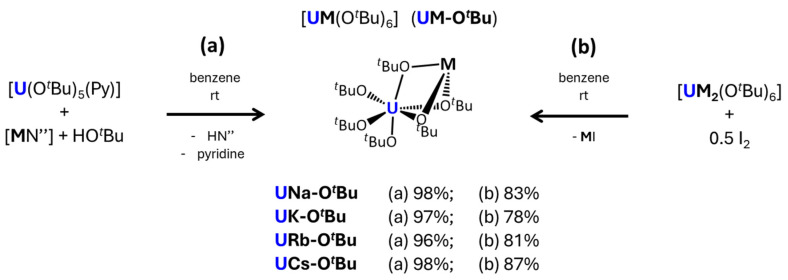
Synthesis of the pentavalent uranium alkali metal *tert*-butoxide [UM(O*^t^*Bu)_6_] (**UM-O*^t^*Bu**) (M = Li, Na, K, Rb) (**a**) by reacting stoichiometric ratios of [U(O*^t^*Bu)_5_(py)], [MN”] (N” = (-N(SiMe_3_)_2_)) and HO*^t^*Bu in benzene at room temperature and (**b**) by oxidizing the tetravalent uranium alkali metal *tert*-butoxides [UM_2_(O*^t^*Bu)_6_] (M = Na, K, Rb, Cs) with elemental iodine. Synthetic route “a” afforded the products in nearly quantitative yields (≥96%), while route “b” gave good yields (78–87%) after filtration to remove the precipitated metal iodide salts.

The U(V)M(I) *tert*-butoxide compounds of the formula [UM(O*^t^*Bu)_6_] (**UM-O*^t^*Bu**-type: **UNa-O*^t^*Bu**, **UK-O*^t^*Bu**, **URb-O*^t^*Bu**, and **UCs-O*^t^*Bu**) were successfully synthesized using two distinct reaction pathways. The first approach involved a straightforward ligand exchange between [U(O*^t^*Bu)_5_(Py)] and [MN”] (M = Na, K, Rb, Cs; N’’ = (-N(SiMe_3_)_2_)) in the presence of HO*^t^*Bu (Scheme 1a). The second route employed oxidative synthesis, wherein the previously reported uranium(IV)-heterometallic alkoxides [UM_2_(O*^t^*Bu)_6_] (M = Na, K, Rb, Cs) were treated with elemental iodine as an oxidizing agent to eliminate alkali metal halides (Scheme 1b).

All U(V)M(I) *tert*-butoxide derivatives were obtained in nearly quantitative yields (≥ 96%) from synthetic route a, as pale golden crystals suitable for single crystal X-ray diffraction, following slow solvent evaporation under an inert argon atmosphere. Additionally, good to very good yields (78–87%) were achieved via synthetic route b, which involved the precipitation of metal iodide salts followed by purification through filtration over a glass wool/celite filter. The isostructural **UM-O*^t^*Bu** compounds crystallized in the trigonal crystal system in the space group *R*3*m* for **UNa-O*^t^*Bu**, **UK-O*^t^*Bu**, and **URb-O*^t^*Bu**, while **UCs-O*^t^*Bu** crystallized in the space group *Immm* (Supplementary Materials Table S1). The crystal structure of **UCs-O*^t^*Bu** exhibited a four-fold packing disorder, with the Cs cation disordered around the [U(O*^t^*Bu)_6_]^−^ octahedron (Supplementary Materials Figure S1). Despite the packing disorder, a suitable structural refinement of **UCs-O*^t^*Bu** was possible, yielding crystallographic reliability factors of R_1_ = 3.29 and wR_2_ = 8.32 that depicted the connectivity of atoms. The molecular structure of the Cs derivative was not used for structural comparison with the other **UM-O*^t^*Bu** derivatives due to the uncertainty in overall structural refinement.

**Figure 3:**
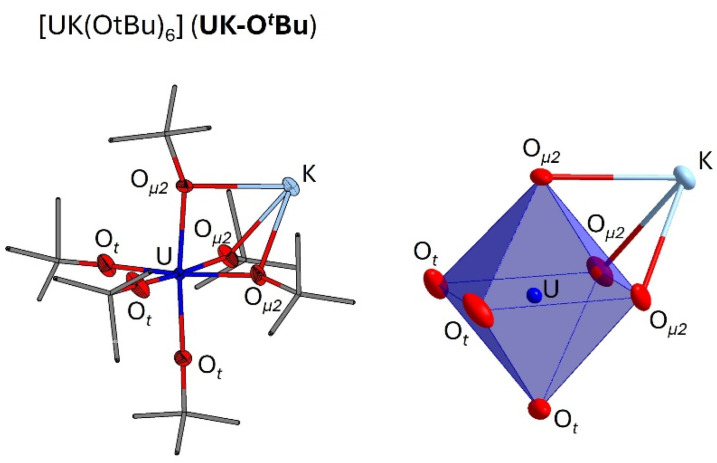
Molecular structure of [UK(O*^t^*Bu)_6_] (**UK-O*^t^*Bu**). Thermal ellipsoids are shown at the 50% probability level and hydrogen atoms have been omitted for clarity.

The uranium(V) centers in the molecular structures of **UM-O*^t^*Bu** (M = Na, K, Rb) were coordinated by six *tert*-butoxide ligands, displaying a distorted octahedron. Unlike the [LiU(O*^t^*Bu)_6_(Et_2_O)] complex reported by Hayton et al. (Figure 1) [16], in which the coordination environment differed significantly, the U(V)M(I) compounds presented in this study exhibited distinct binding motifs, where three ligands bound terminally to the U(V) center, while the remaining three adopted a *μ*_2_-bridging mode to the alkali metal M(I) cation (Figure 3 for **UK-O*^t^*Bu**). The molecular structure featured a trigonal bipyramidal [UMO_3_] framework, where one uranium center, one alkali metal center, and three *μ*_2_-bridging *tert*-butoxide ligands occupied the vertices of the trigonal bipyramid.

**Figure 4:**
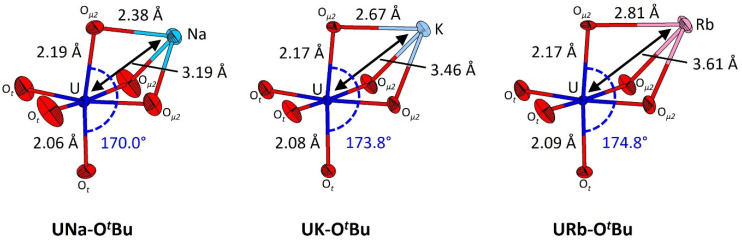
The AnMO_6_ frameworks of the [AnM(O*^t^*Bu)_6_] derivatives (**UM-O*^t^*Bu**-type (M = Na, K, Rb)) with the corresponding An–M distances, the O_t_–An–O*_μ_*_2_ (trans) angles, and the An–O*_t_* and An–O*_μ2_* bond lengths.

The selected U–O bond length and angles in **UNa-O*^t^*Bu**, **UK-O*^t^*Bu**, and **URb-O*^t^*Bu** derivatives were comparable to the previously reported uranium(V) *tert*-butoxides [ULi(O*^t^*Bu)_6_(Et_2_O)] [16], [U(O*^t^*Bu)_5_(Py)] [14], [U_2_(O*^t^*Bu)_9_] [15], and [UM(O*^t^*Bu)_7_] (M^II^ = Ge, Sn, Pb) [14]. In particular, the U–O*_t_* (2.06 Å–2.09 Å) and U–O*_µ_*_2_ bond lengths (2.17 Å–2.19 Å) agreed with the reported values for the terminal (2.05 Å–2.08 Å) and doubly bridged U(V)–O (*tert*-butoxide) (2.237(13) Å) bond lengths [14,15,16]. Comparative analysis of **UNa-O*^t^*Bu**, **UK-O*^t^*Bu**, and **URb-O*^t^*Bu** showed that the size of the alkali metal ion influenced the U–M distance, with a gradual increase in bond distance with increasing size of the alkali metal cation (3.19 Å **UNa-O*^t^*Bu**; 3.46 Å **UK-O*^t^*Bu**; 3.61 Å **URb-O*^t^*Bu**) (Figure 4).

This trend was attributed to increased steric repulsion between the metal centers and alkoxo-ligands, leading to an elongation of the M–O*_µ_*_2_ bond length, while the U–O*_µ_*_2_ contacts remained constant throughout the U(V)M(I) *tert*-butoxide series. However, the O*_µ_*_2_–U–O*_t_ trans* bond angles indicated greater distortion of the U(V) octahedron in derivatives with smaller alkali metals, which was similar to the findings we previously reported on the influence of alkali metal cations on the formation of the heterobimetallic actinide(IV) *tert*-butoxides [AnM_3_(O*^t^*Bu)_7_] and [AnM_2_(O*^t^*Bu)_6_] (An = Th, U, M = Li, Na, K, Rb, Cs) [46]. Also here, increased distortion correlated with a slight increase in U–O*_µ_*_2_ bond length and a decrease in U–O*_t_* bond length, which was attributed to steric repulsion. A benzene ring located near the alkali metal appeared to be a solvent incorporation and not π-coordination, as indicated by its relatively large distance from the alkali metal, measuring 3.29 Å or more. Similarly, a benzene ring was found to be located near one of the alkali metals in the reported [AnM_2_(O*^t^*Bu)_6_] (An = Th, U, M = Li, Na, K, Rb, Cs) derivatives [46].

**Figure 5:**
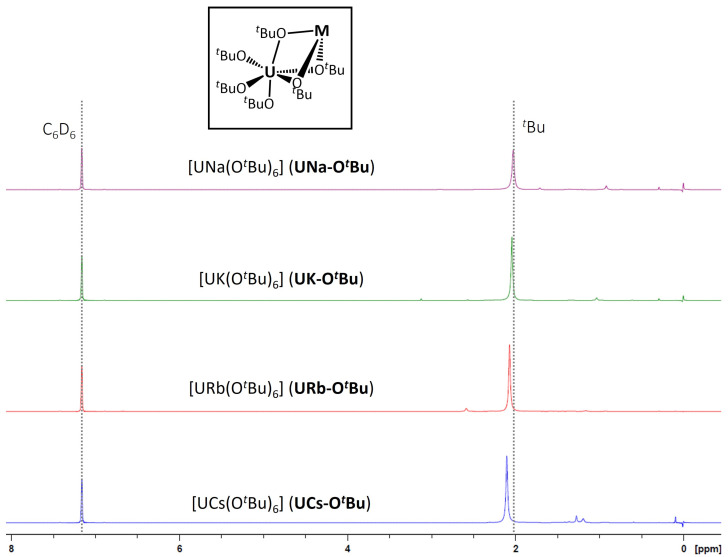
^1^H NMR comparison of the heterobimetallic U^V^-M^I^ *tert*-butoxide complexes [UM(O^t^Bu)_6_] (**UM-O*^t^*Bu**-type: **UNa-O*^t^*Bu**, **UK-O*^t^*Bu**, **URb-O*^t^*Bu**, and **UCs-O*^t^*Bu**) measured on a 300 MHz Bruker Avance 300 spectrometer in benzene-d*_6_*. For comparison, the position of the benzene-*d_6_* signal and the *tert*-butoxide (*^t^*Bu) resonance of **UNa-O*^t^*Bu** are added as dashed reference lines. Minor impurity signals may have originated from residual protonated ligands such as HO*^t^*Bu and HN’’ or from small amounts of unreacted starting materials, including [U(O*^t^*Bu)_5_(py)] [14] and [MN’’] (M = Na–Cs) [47,48].

The ^1^H NMR spectra of the U(V)M(I) bimetallic compounds (**UNa-O*^t^*Bu**, **UK-O*^t^*Bu**, **URb-O*^t^*Bu**, and **UCs-O*^t^*Bu**) in benzene-*d_6_* showed single resonances at 2.03, 2.05, 2.07, and 2.10 ppm, respectively, for the 6 *tert*-butoxide ligands surrounding the uranium(V) center (Figure 5 and Supplementary Materials Figures S3–S6). The differentiation between terminal and bridging alkoxide ligands was not possible in solution, indicating a dynamic coordination environment in **UM-O*^t^*Bu** derivatives. This behavior may have indicated a fluxional structure with potential alkali metal cation exchange between coordination sites of the [U(O*^t^*Bu)_6_]^−^ octahedron, analogous to previously reported solution dynamics in actinide-alkali metal structures [16,46]. However, increasing the alkali metal size from Na to Cs resulted in a slight downfield shift (δ 2.03 ppm to δ 2.10 ppm), which reflected subtle changes in the bonding and chemical environments in solution.

The **UM-O*^t^*Bu**-type structures [UM(O*^t^*Bu)_6_] were further characterized by IR spectroscopy, revealing band patterns similar to the data previously reported by our group on heterobimetallic actinide(IV) alkali metal alkoxides [AnM_2_(O*^t^*Bu)_6_] and [AnM_3_(O*^t^*Bu)_7_] (Supplementary Materials Figure S2) [46].

### 2.2. U(V)M(I) iso-Propoxide Compounds

**Scheme 2:**
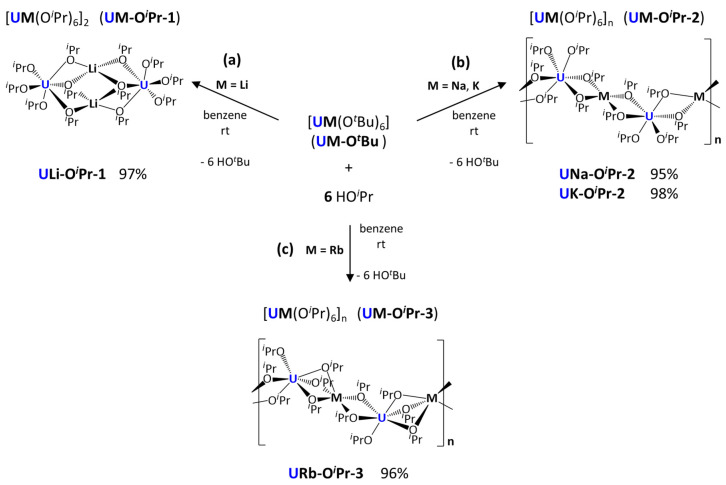
Synthesis of the mixed-metal alkali metal uranium *iso*-propoxides [UM(O*^i^*Pr)_6_]*_n_* ((**a**) *n* = 2, M = Li (**ULi-O*^i^*Pr-1**); (**b**) *n* = ∞, M = Na (**UNa-O*^i^*Pr-2**), K (**UK-O*^i^*Pr-2**); (**c**) *n* = ∞, M = Rb (**URb-O*^i^*Pr-3**)) in nearly quantitative yields (≥96%) by trans-alcoholysis of the *tert*-butoxide compounds [ULi(O*^t^*Bu)_6_(Et_2_O)] [16] **UNa-O*^t^*Bu**, **UK-O*^t^*Bu**, and **URb-O*^t^*Bu** with *iso*-propyl alcohol.

The U(V)M(I) *iso*-propoxide derivatives of the general formula [UM(O*^i^*Pr)_6_]*_n_* (*n* = 2, M = Li (**ULi-O*^i^*Pr-1**); *n* = ∞, M = Na (**UNa-O*^i^*Pr-2**), K (**UK-O*^i^*Pr-2**), Rb (**URb-O*^i^*Pr-3**)) were synthesized via trans-alcoholysis of the *tert*-butoxide compounds [ULi(O*^t^*Bu)_6_(Et_2_O)] [16] (**ULi-Et_2_O**) and [UM(O*^t^*Bu)_6_] (**UM-O*^t^*Bu**-type: **UNa-O*^t^*Bu**, **UK-O*^t^*Bu**, and **URb-O*^t^*Bu**) with *iso*-propyl alcohol (Scheme 2).

Slow evaporation of the solvent produced nearly quantitative yields (≥96%) of colorless crystals of the **UM-O*^i^*Pr** derivatives. The crystallographic data are provided in Supplementary Materials Table S2. In contrast to the *tert*-butoxide derivatives, coordination of the less sterically demanding *iso*-propoxide ligands led to oligomerization of the resulting U(V)M(I) *iso*-propoxide compounds. Depending on the ionic radius of the alkali metal cations, three distinct structural motifs were identified. For example, the smallest alkali metal cation lithium (0.76 Å (c.n.6)) afforded a dimeric **UM-O*^i^*Pr-1** alkoxide [UM(O*^i^*Pr)_6_]_2_ (M = Li) (Scheme 2a), whereas the larger sodium (1.02 Å) and potassium (1.38 Å) ions resulted in the formation of the polymeric **UM-O*^i^*Pr-2**-type structures [UM(O*^i^*Pr)_6_]_∞_ (M = Na, K), which contained fourfold-coordinated alkali metal centers (Scheme 2b). By contrast, a fivefold-coordinated rubidium center was observed for the significantly larger Rb^+^ (1.52 Å) present in the polymeric structure [UM(O*^i^*Pr)_6_]_∞_ (**UM-O*^i^*Pr-3**; M = Rb) (Scheme 2c).

### 2.3. Dimeric [UM(O^i^Pr)_6_]_2_ Derivatives

**Figure 6:**
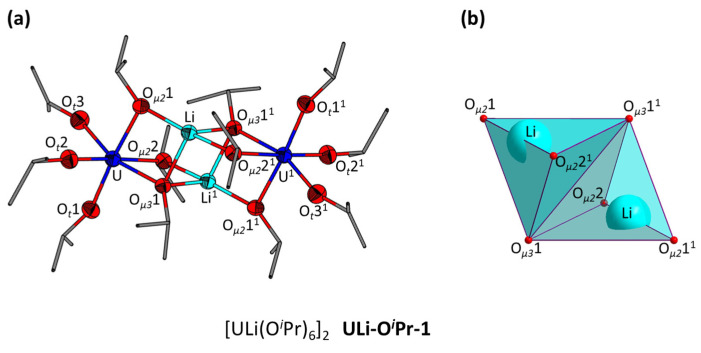
(**a**) Molecular structure of [ULi(O*^i^*Pr)_6_]_2_ (**ULi-O*^i^*Pr-1**). Thermal ellipsoids are shown at the 50% probability level and hydrogen atoms have been omitted for clarity. (**b**) Edge-sharing tetrahedral geometries of *iso*-propoxide ligands surrounding the Li^I^ cations. Selected interatomic distances [Å], bond distances [Å], and bond angles [°]: U–M 3.18(1), M–M 2.65(1); U–O*_t_*1 2.043(5), U–O*_t_*2 2.044(5), U–O*_t_*3 2.121(5), U–O*_µ_*_2_1 2.238(4), U–O*_µ_*_2_2 2.223(4), U–O*_µ_*_3_1 2.192(4), Li–O*_µ_*_2_1 1.941(8), Li–O*_µ_*_3_1 2.052(7), Li–O*_µ_*_2_2^1^ 2.010(8), Li–O*_µ_*_3_1^1^ 2.070(7); O*_t_*1–U–O*_µ_*_2_1 175.5(2), O*_t_*2–U–O*_µ_*_2_2 161.2(2), O*_t_*3–U–O*_µ_*_2_3 175.6 (2), O*_µ_*_2_1–U–O*_µ_*_3_1 76.7(2), O*_µ_*_2_1–U–O*_µ_*_2_2 90.2(2), O*_µ_*_2_2–U–O*_µ_*_3_1 76.7(2), O*_µ_*_2_1–Li–O*_µ_*_2_2^1^ 164.6(4), O*_µ_*_2_1–Li–O*_µ_*_3_1 86.5(3), O*_µ_*_3_1–Li–O*_µ_*_3_1^1^ 100.2(3), and O*_µ_*_3_1–Li–O*_µ_*_2_2^1^ 100.8(2).

The central core of the dimeric [ULi(O*^i^*Pr)_6_]_2_ (**ULi-O*^i^*Pr-1**) resembled a [U_2_Li_2_O_6_] cage (Figure 6a) formed by two analogous seco-norcubane subunits sharing a common face defined by a Li_2_O_2_ ring. Similar [M_2_Li_2_O_6_] seco-norcubane-type structures have been reported for the dimeric *iso*-propoxide complexes [MLi(O*^i^*Pr)_5_]_2_ (M = Ti [49], Hf [50]). **ULi-O*^i^*Pr-1** contained two distorted-octahedral uranium(V) centers connected by lithium bridges. Each U(V) center possessed three terminal, two *µ*_2_-bridging, and one *µ*_3_-bridging *iso*-propoxide ligands in the coordination sphere. The Li(I) cations were found in edge-connected, highly distorted tetrahedral coordination environments surrounded by four *iso*-propoxide (2 × O*_µ_*_2_, 2 × O*_µ_*_3_) ligands each (Figure 6b).

The U–Li distance of 3.18(1) Å lay within the range of previously reported U(IV/V)–Li contacts, including as those in [LiU(O*^t^*Bu)_6_(Et_2_O)] [16] (3.07 Å), [Li_2_U(O*^t^*Bu)_6_(THF)_2_] [16], (2.87 Å) or [ULi_3_(O*^t^*Bu)_7_] [46] (3.24 Å). The steric repulsion between the lithium cations resulted in a relatively short Li–Li distance (2.65(1) Å), placing the metal centers near the O*_µ_*_2_1–O*_µ_*_2_2^1^ or O*_µ_*_2_1^1^–O*_µ_*_2_2 vectors within their respective tetrahedral coordination environments.

The coordination to the lithium(I) cations forced the respective *iso*-propoxide ligands to tilt toward each other within the [U(O*^i^*Pr)_6_] octahedron, as evident in the small O*_µ_*_2_1–U–O*_µ_*_3_1 and O*_µ_*_2_2–U–O*_µ_*_3_1 angles (76.7°). Furthermore, this interaction led to bond length elongation in bridged U–OR units (U–O*_µ_*_2_1 2.238(4) Å, U–O*_µ_*_2_2 2.223(4) Å, U–O*_µ_*_3_1 2.192(4) Å), when compared to the terminal alkoxide ligands (U–O*_t_*1 2.043(5) Å, U–O*_t_*2 2.044(5) Å, U–O*_t_*3 2.121(5) Å). The U–O*_t_* bond lengths were found to be in good agreement with other reported U(V) –O (alkoxide) bond lengths (2.03–2.08 Å) [14,15,16]. The same applied to the lithium(I) –oxygen bonds, which were comparable to previously reported Li(I) –O (alkoxide) bond length [16,46,51].

### 2.4. Polymeric [UM(O^i^Pr)_6_]_∞_ Derivatives

**Figure 7:**
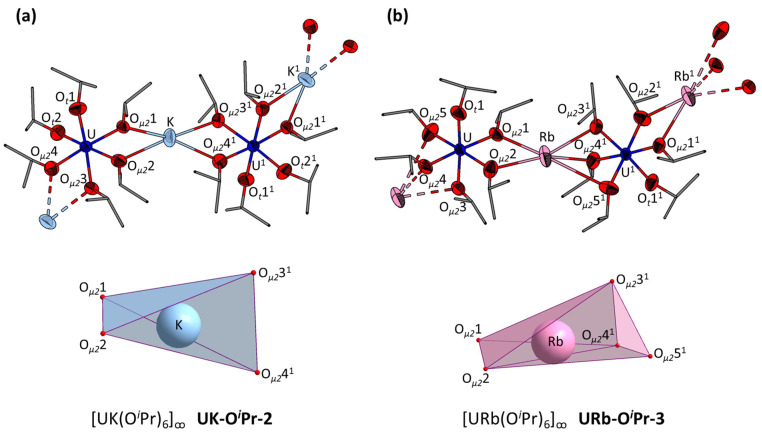
Molecular structure of (**a**) (**UK-O*^i^*Pr-2**) and (**b**) (**URb-O*^i^*Pr-3**). Thermal ellipsoids are shown at the 50% probability level and hydrogen atoms have been omitted for clarity.

The [UM(O*^i^*Pr)_6_]_∞_ derivatives based on larger alkali metal cations produced **UM-O*^i^*Pr-2**-type (M = Na, K) and **UM-O*^i^*Pr-3**-type (M = Rb) polymeric structures composed of interconnected [UMO_6_] units (Figure 7a,b). In the case of the **UM-O*^i^*Pr-2** derivatives, four alkoxide ligands of the U(V)-octahedron were doubly bridged (O*_µ_*_2_) to the neighboring alkali metals, whereas the U(V) center in the **UM-O*^i^*Pr-3**-type possessed five *µ*_2_-bridging *iso*-propoxide ligands. Consequently, the alkali metal centers were present in distorted tetrahedral (Figure 7a) or highly distorted square pyramidal coordination environments (Figure 7b), respectively.

Selected, uranium–metal distances, bond lengths, and angles are summarized in Table 1 Compared to the previously reported U(V)M(I) *tert*-butoxide derivatives [UM(O*^t^*Bu)_6_] (**UM-O*^t^*Bu-**type: **UNa-O*^t^*Bu**, **UK-O*^t^*Bu**, and **URb-O*^t^*Bu**), the uranium–metal distances in the *iso*-propoxide series were elongated by ~0.4 Å. This increase was attributed to the polymeric, bidentate coordination mode of the [U(O*^i^*Pr)_6_] octahedra, which bridged two alkali metal centers. Notably, in [URb(O*^i^*Pr)_6_]_∞_ (**UM-O*^i^*Pr-3**), a significant reduction in the U–Rb distance was observed at the site with tridentate U–Rb contact (3.781(1) Å) when compared to the bidentate-coordinated site (U–Rb: 4.009(1) Å).

For the sodium, potassium, and rubidium iso-propoxide derivatives, the U–O*_t_* bond lengths were consistent with previously reported U(V)–O (alkoxide) bond distances [14,15,16]. A comparison of the trans O*_t_*1–U–O*_µ_*_2_3 and cis O*_µ_*_2_1–U–O*_µ_*_2_2 bond angles indicated a greater distortion of the octahedral geometry in derivatives containing smaller alkali metals. Conversely, the coordination polyhedron around the alkali metals showed a higher distortion for derivatives with larger alkali metals, as reflected by the narrower O*_µ_*_2_1–M–O*_µ_*_2_2 angles and longer M–O*_µ_*_2_ distances. Furthermore, the presence of an additional bridging alkoxide ligand (O*_µ_*_2_5) in [URb(O*^i^*Pr)_6_]_∞_ (UM-O*^i^*Pr-3) derivative resulted in an elongated Rb–O*_µ_*_2_5 bond of 3.438 Å, significantly longer than the other Rb–O*_µ_*_2_ bonds (2.808–2.894 Å), indicating weaker coordination. Nonetheless, a weak interaction could be discerned based on the relatively elongated U–O*_µ_*_2_5 bond (2.105 Å) when compared to the U–O*_t_*1 bond of the terminal alkoxide ligand (2.077(5) Å).

The room-temperature ^1^H NMR spectra of the uranium(V)-alkali metal *iso*-propoxides showed high complexity, with respect to the signal assignment based on the solid-state structures. All spectra exhibited 4 to 6 resonances in the range of 1.06 to 12.70 ppm (Supplementary Materials Figures S7–S10). A similar observation was reported for the alkali metal uranium(IV) *tert*-butoxides [ULi_3_(O*^t^*Bu)_7_] and [UM_2_(O*^t^*Bu)_6_] and was attributed to the paramagnetism arising from the 5f¹ electronic configuration of uranium(V), accompanied by the dynamic behavior of the alkali metal cations in solution [46]. However, all of the [UM(O*^i^*Pr)_6_]*_n_* derivatives (*n* = 2, M = Li; *n* = ∞, M = Na, K, Rb) displayed intense NMR resonances between 1.90 and 2.07 ppm.

The **UM-O*^i^*Pr** derivatives were further characterized using IR spectroscopy (Supplementary Materials Figure S11). The recorded spectra exhibited comparable peak patterns across the series. In comparison to the IR spectra of uranium(V) *tert*-butoxides discussed in previous sections, the primary difference lay in the ν(C–C) skeletal stretching vibration band. This band appeared at 1125 cm^−1^ for the *iso*-propoxide derivatives, whereas it was observed at 1180 cm^−1^ for the *tert*-butoxides.

## 3. Experimental Section

Given that U(V)-alkoxides are generally sensitive to hydrolysis and oxidation upon exposure to air at ambient conditions, all reactions were performed under inert conditions in a glovebox under an argon atmosphere and less than 0.1 ppm H_2_O and O_2_ or using standard Schlenk techniques. Chemicals were obtained from Sigma-Aldrich Chemical Co. (Saint Louis, MO, USA), Acros Organics (Geel, Belgium), Alfa Aesar (Ward Hill, MA, USA), VWR (Singapore), Fisher Scientific (Waltham, MA, USA), and Strem Chemical Inc. (Edmonton, AB, Canada). Solvents and deuterated solvents were distilled prior to use and were dried and stored over potassium under an argon atmosphere. Tert-butyl alcohol and iso-propyl alcohol were dried by refluxing over CaH_2_ for 2 days. All solvents and deuterated solvents were degassed using the freeze-pump-thaw technique, brought into the glovebox, and stored over dried molecular sieves (3 Å).

[U(O*^t^*Bu)_5_(py)] [14], [ULi_2_(O*^t^*Bu)_6_(THF)_2_] [16], [UM_2_(O*^t^*Bu)_6_] [46], and the alkali metal silyl amides [MN”] (M^I^ = Li [52], Rb [47], Cs [48]) were synthesized according to literature methods and used as starting materials for the heterobimetallic uranium(V)-alkali metal(I) *tert*-butoxides [UM(O*^t^*Bu)_6_] (M = Na, K, Rb, Cs). The alkali metal silyl amides [MN”] (M^I^ = Na, K) were purchased from Sigma Aldrich. According to German legislation, the natural uranium/thorium used in this work was not classified as “radioactive” but merely as a chemical element in its natural isotopic composition, since the total inventories of natural uranium and thorium in the laboratory did not exceed 100 g and 200 g, respectively. Hence, no additional precautions were necessary, and the uranium/thorium precursors were handled and stored taking similar precautions to those applicable to any hazardous heavy metal compound.

For single-crystal X-ray diffraction (SXRD) analysis, several as-grown single crystals were placed on a microscope slide and covered with Fomblin YR-1800 oil (Thermo Scientific, Waltham, MA, USA) inside the glovebox. The prepared slide was sealed in a 50 mL Falcon^®^ centrifuge tube (Corning Inc, Corning, NY, USA) under an inert argon atmosphere and transferred to the SXRD instrument. A suitable single crystal was then selected, mounted on a MiTiGen MicroLoop^TM^ (MiTeGen, Ithaca, NY, USA), and affixed to the goniometer head of a Bruker D8 Venture diffractometer (Billerica, MA, USA). The crystal was cooled to 100–120 K using an Oxford Cryostream low-temperature device (Oxford Cryosystems Ltd., Oxford, UK). The full dataset was recorded, and the images were processed using APEX2 (Bruker AXS Inc., Billerica, MA, USA). Structure solution by direct methods was achieved using SHELXS programs, and the structural model was refined by full matrix least squares on F 2 using SHELX97 (G. M. Sheldrick, University of Göttingen, Göttingen, Germany). Molecular graphics were plotted using Diamond (Crystal Impact, Bonn, Germany). Editing of CIFs and construction of tables, bond lengths, and angles were achieved using PLATON (A.L. Spek—Version 60521, Utrecht University, Utrecht, The Netherlands) and Olex2 (OlexSys Ltd., Durham, UK). The NMR spectra were recorded on a Bruker Avance 300 spectrometer (Bruker BioSpin GmbH, Rheinstetten, Germany), in freshly distilled and degassed benzene-*d_6_* and pyridine-*d_5_* at 298 K using NMR-tubes with J-Young valve (Wilmad-LabGlass, Vineland, NJ, USA). The ^1^H NMR spectra (300.13 MHz) were acquired using the pulse sequence zg30 with 64 scans, a spectral width of 199.51 ppm (corresponding to a chemical shift range of approximately ±100 ppm), and a relaxation delay of 1.20 s. Chemical shifts are reported in parts per million (ppm) relative to external tetramethylsilane and are referenced internally to the proton impurity of the solvent. For characterization of the observed signal multiplicities, the following abbreviations were used: s (singlet), m (multiplet), as well as br (broad). The NMR spectra were analyzed using the software Bruker Topspin 4.1.1 (Bruker BioSpin GmbH, Rheinstetten, Germany). Infrared spectra were obtained using a Platinum ATR spectrometer (Bruker Optik GmbH, Ettlingen, Germany) on a crystal plate, with the samples analyzed using OPUS software (Version 7.8, Bruker Optik GmbH, Ettlingen, Germany). The spectrometer was placed in an argon glovebox. Elemental analyses were carried out using a HEKAtech CHNS Euro EA 3000 (HEKAtech GmbH, Wegberg, Germany). The sample preparation was performed in a glove box by weighing 1–2 mg product in a tin cartridge. To protect the product from oxidation, the folded cartridge was sheathed with another tin cartridge, since the reweighing of the cartridges was performed outside under an environmental atmosphere. Deviation of the CHNS data from the calculated values could be attributed to the extraordinary sensitivity of the compounds.

### 3.1. General Reaction Procedure for [UM(O^t^Bu)_6_] (UM-O^t^Bu)

(a) Co-Alcoholysis Reaction Starting From the U^V^ *tert*-Butoxide [U(O*^t^*Bu)_5_(Py)]. In a 5 mL snap-cap vial, a mixture of 1.0 eq. [U(O*^t^*Bu)_5_(py)] and 1.0 eq. [M(N(SiMe_3_)_2_)] was dissolved in 1 mL benzene, followed by the addition of 1.0 eq. *tert*-butanol dissolved in 1 mL benzene. The mixture was stirred for 1 d at room temperature in a closed vial. Afterward, the vial was removed from the stir plate and stored with the cap closed. Slow evaporation of all volatiles at room temperature yielded crystals of [AnM(O*^t^*Bu)_6_] (**AnM-O*^t^*Bu**).
(b) Redox Reaction Starting from the U^IV^ *tert*-Butoxides [UM_2_(O*^t^*Bu)_6_]. In a 5 mL snap-cap vial, 1.0 eq. [UM_2_(O*^t^*Bu)_6_] was reacted with 0.5 eq. I_2_ in benzene. The mixture was stirred for 1 d at room temperature in a closed vial, resulting in the precipitation of MI. After filtration through celite/glass wool and subsequent slow evaporation of all volatiles at room temperature in a closed snap-cap vial, crystals of [AnM(O*^t^*Bu)_6_] (**AnM-O*^t^*Bu**) were obtained.

Synthesis of [UNa(O*^t^*Bu)_6_] (UNa-O*^t^*Bu). (a) 15.0 mg (22.3 µmol) of [U(O*^t^*Bu)_5_(py)], 4.1 mg (22.3 µmol) [Na(N(SiMe_3_)_2_)], and 1.7 mg (22.3 µmol) of *tert*-butanol were reacted to give [UNa(O*^t^*Bu)_6_] (**UNa-O*^t^*Bu**) in the form of colorless crystals in a nearly quantitative yield of 15.3 mg (98%). (b) 30.0 mg (41.5 µmol) of [UNa_2_(O*^t^*Bu)_6_] was reacted with 5.3 mg (20.8 µmol) I_2_ to give [UNa(O*^t^*Bu)_6_] (**UNa-O*^t^*Bu**) in the form of colorless crystals in a yield of 24.1 mg (83%). ^1^H NMR (300 MHz, 25 °C, C_6_D_6_): δ 2.03 (terminal U-OC(C*H*_3_)_3_, s, 54H). IR (cm^−1^): 2973 (m), 2867 (w), 1463 (w), 1382 (w), 1357 (m), 1231 (m), 1185 (s), 951 (s), 769 (w), 693 (w), 486 (s).

Synthesis of [UK(O*^t^*Bu)_6_] (UK-O*^t^*Bu). (a) 15.0 mg (22.3 µmol) of [U(O*^t^*Bu)_5_(py)], 4.4 mg (22.3 µmol) [K(N(SiMe_3_)_2_)], and 1.7 mg (22.3 µmol) of *tert*-butanol were reacted to give [UK(O*^t^*Bu)_6_] (**UK-O*^t^*Bu**) in the form of colorless crystals in a nearly quantitative yield of 15.5 mg (97%). (b) 30.0 mg (39.7 µmol) of [UK_2_(O*^t^*Bu)_6_] was reacted with 5.0 mg (19.9 µmol) I_2_ to give [UK(O*^t^*Bu)_6_] (**UK-O*^t^*Bu**) in the form of colorless crystals in a yield of 22.2 mg (78%). ^1^H NMR (300 MHz, 25 °C, C_6_D_6_): δ 2.05 (terminal U-OC(C*H*_3_)_3_, s, 54H). IR (cm^−1^): 2967 (m), 2867 (w), 1461 (w), 1382 (w), 1357 (m), 1226 (m), 1180 (s), 947 (s), 769 (w), 688 (w), 486 (s).

Synthesis of [URb(O*^t^*Bu)_6_] (URb-O*^t^*Bu). (a) 15.0 mg (22.3 µmol) of [U(O*^t^*Bu)_5_(py)], 5.5 mg (22.3 µmol) [Rb(N(SiMe_3_)_2_)], and 1.7 mg (22.3 µmol) of *tert*-butanol were reacted to give [URb(O*^t^*Bu)_6_] (**URb-O*^t^*Bu**) in the form of colorless crystals in a nearly quantitative yield of 16.3 mg (96%). (b) 30.0 mg (35.4 µmol) of [URb_2_(O*^t^*Bu)_6_] was reacted with 4.5 mg (17.7 µmol) I_2_ to give [URb(O*^t^*Bu)_6_] (**URb-O*^t^*Bu**) in the form of colorless crystals in a yield of 21.9 mg (81%). ^1^H NMR (300 MHz, 25 °C, C_6_D_6_): δ 2.07 (terminal U-OC(C*H*_3_)_3_, s, 54H). IR (cm^−1^): 2972 (m), 2867 (w), 1463 (w), 1382 (w), 1357 (m), 1226 (m), 1180 (s), 947 (s), 769 (w), 486 (s).

Synthesis of [UCs(O*^t^*Bu)_6_] (UCs-O*^t^*Bu). (a) 15.0 mg (22.3 µmol) of [U(O*^t^*Bu)_5_(py)], 6.5 mg (22.3 µmol) [Cs(N(SiMe_3_)_2_)], and 1.7 mg (22.3 µmol) of *tert*-butanol were reacted to give [UCs(O*^t^*Bu)_6_] (**UCs-O*^t^*Bu**) in the form of colorless crystals in a nearly quantitative yield of 17.7 mg (98%). (b) 30.0 mg (31.8 µmol) of [UCs_2_(O*^t^*Bu)_6_] was reacted with 4.0 mg (15.9 µmol) I_2_ to give [UCs(O*^t^*Bu)_6_] (**UCs-O*^t^*Bu**) in the form of colorless crystals in a yield of 22.4 mg (87%). ^1^H NMR (300 MHz, 25 °C, C_6_D_6_): δ 2.11 (terminal U-OC(C*H*_3_)_3_, s, 54H). IR (cm^−1^): 2977 (m), 2867 (w), 1468 (w), 1382 (w), 1357 (m), 1226 (m), 1180 (s), 941 (s), 769 (w), 486 (s).

### 3.2. General Reaction Procedure for [UM(O^i^Pr)_6_]_n_ (UM-O^i^Pr)

In a 5 mL snap-cap vial, 1.0 eq. [UM(O*^t^*Bu)_6_] (**UM-O*^t^*Bu**) was reacted with 6.0 eq. *iso*-propanol in 2 mL benzene in a closed vial. The mixture was stirred for 1 d at room temperature. Afterward, the vial was removed from the stir plate and stored with the cap closed. After slow evaporation of all volatiles, crystals of [UM(O*^i^*Pr)_6_]_n_ (**UM-O*^i^*Pr**) were obtained.

Synthesis of [ULi(O*^i^*Pr)_6_]_2_ (ULi-O*^i^*Pr-1). 15.0 mg (19.8 µmol) [ULi(O*^t^*Bu)_6_(Et_2_O)] (**ULi-Et_2_O**) was reacted with 7.1 mg (118.8 µmol) *iso-*propanol to give [ULi(O*^i^*Pr)_6_]_2_ (**ULi-O*^i^*Pr-1**) in the form of colorless crystals in a nearly quantitative yield of 11.5 mg (97%). ^1^H NMR (300 MHz, 25 °C, C_5_D_5_N): δ 12.65 (b), δ 1.90 (m), δ 1.40 (s), δ 1.32 (m). IR (cm^−1^): 2975 (m), 2936 (w), 2873 (w), 1463 (w), 1360 (m), 1232 (w), 1165 (m), 1122 (s), 1000 (m), 952 (s), 826 (s), 778 (m), 543 (s), 506 (s), 467 (m), 406 (s). Anal. Calcd. U_2_Li_2_O_12_C_36_H_84_: C 39.06, H 7.06. Found: C 37.87, H 7.24.

Synthesis of [UNa(O*^i^*Pr)_6_]_∞_ (UNa-O*^i^*Pr-2). 15.0 mg (21.4 µmol) [UNa(O*^t^*Bu)_6_] (**UNa-O*^t^*Bu**) (699.70) was reacted with 7.7 mg (128.4 µmol) *iso-*propanol to give [UNa(O*^i^*Pr)_6_]_∞_ (**UNa-O*^i^*Pr-2**) in the form of colorless crystals in a nearly quantitative yield of 12.5 mg (95%). ^1^H NMR (300 MHz, 25 °C, C_5_D_5_N): δ 12.52 (b), δ 7.37 (s), δ 2.05 (m), δ 1.42 (s). IR (cm^−1^): 2969 (m), 2930 (w), 2867 (w), 1453 (w), 1360 (m), 1331 (w), 1229 (w), 1183 (m), 1159 (m), 1120 (s), 1002 (w), 955 (s), 825 (m), 775 (m), 700 (w), 535 (s), 505 (s), 442 (m), 409 (s). Anal. Calcd. UNaO_6_C_18_H_42_: C 35.12, H 6.88. Found: C 38.81, H 7.47.

Synthesis of [UK(O*^i^*Pr)_6_]_∞_ (UK-O*^i^*Pr-2). 15.0 mg (21.0 µmol) [UK(O*^t^*Bu)_6_] (**UK-O*^t^*Bu**) was reacted with 7.6 mg (126.0 µmol) *iso-*propanol to give [UK(O*^i^*Pr)_6_]_∞_ (**UK-O*^i^*Pr-2**) in the form of colorless crystals in a nearly quantitative yield of 13.0 mg (98%). ^1^H NMR (300 MHz, 25 °C, C_5_D_5_N): δ 12.64 (b), δ 7.37 (s), δ 2.52 (m), δ 2.07 (m), δ 1.39 (s), δ 1.32 (m). IR (cm^−1^): 2967 (m), 2933 (w), 2869 (w), 1466 (w), 1359 (m), 1331 (w), 1235 (w), 1180 (m), 1158 (m), 1120 (s), 1002 (w), 949 (s), 830 (s), 777 (m), 672 (w), 533 (s), 503 (s), 445 (m), 408 (s). Anal. Calcd. UKO_6_C_18_H_42_: C 34.23, H 6.70. Found: C 35.28, H 6.83.

Synthesis of [URb(O*^i^*Pr)_6_]_∞_ (URb-O*^i^*Pr-3). 15.0 mg (19.7 µmol) [URb(O*^t^*Bu)_6_] (**URb-O*^t^*Bu**) was reacted with 7.1 mg (118.2 µmol) *iso-*propanol to give [URb(O*^i^*Pr)_6_]_∞_ (**URb-O*^i^*Pr-3**) in the form of colorless crystals in a nearly quantitative yield of 14.4 mg (96%). ^1^H NMR (300 MHz, 25 °C, C_5_D_5_N): δ 12.70 (b), δ 7.37 (s), δ 2.52 (m), δ 2.07 (m), δ 1.40 (s), δ 1.06 (s). IR (cm^−1^): 2971 (m), 2931 (w), 2865 (w), 1459 (w), 1360 (m), 1331 (w), 1229 (w), 1186 (m), 1159 (m), 1122 (s), 1002 (w), 952 (s), 830 (s), 776 (m), 700 (w), 529 (s), 504 (s), 442 (m), 405 (s). Anal. Calcd. UNaO_6_C_18_H_42_: C 31.89, H 6.24. Found: C 33.80, H 6.50.

## 4. Conclusions

In summary, this study demonstrates that pentavalent uranium can be readily stabilized in combination with varying monovalent alkali metals through the formation of well-defined *tert*-butoxide and *iso*-propoxide complexes. Heterobimetallic uranium(V) alkoxides of the general formula [UM(O*^t^*Bu)_6_] (**UM-O*^t^*Bu**; M = Na, K, Rb, Cs) were successfully synthesized by two complementary routes based on: (i) co-alcoholysis of the uranium(V) alkoxide [U(O*^t^*Bu)_5_(py)] with alkali metal silylamides and *tert*-buyl alcohol, and (ii) oxidative conversion of the corresponding uranium(IV) *tert*-butoxides [UM_2_(O*^t^*Bu)_6_] (M = Na, K, Rb, Cs) using elemental iodine. Subsequent trans alcoholysis reactions with *iso*-propyl alcohol yielded a series of uranium(V)-alkali metal *iso*-propoxides [UM(O*^i^*Pr)_6_]*_n_* (**UM-O*^i^*Pr**; *n* = 2, M = Li; *n* = ∞, M = Na, K, Rb) that exhibited three distinct structural motifs. Notably, the coordination environments of the alkali metal centers varied significantly across the series, reflecting the influence of ionic size and steric effects on the structural assembly. In summary, the findings reported here demonstrate new synthetic pathways for accessing a structurally diverse family of heterobimetallic uranium(V)-alkali metal alkoxides. These complexes not only broaden the structural diversity of uranium coordination chemistry but also hold significant promise as synthetic precursors in salt metathesis reactions for the targeted synthesis of new heterobimetallic uranium alkoxides. Furthermore, they are promising precursors for the development of bimetallic uranium oxides, which are of particular interest for applications such as solar-assisted hydrogen generation [53,54] and next-generation battery materials [55] owing to the enhanced electronic properties and rich redox chemistry of uranium centers [3].

## Figures and Tables

**Table 1:** Selected intermetallic distances [Å], bond distances [Å], and bond angles [°] for the **UM-O*^i^*Pr-2**-type [UM(O*^i^*Pr)_6_]_∞_ (M = Na, K) and **UM-O*^i^*Pr-3**-type [UM(O*^i^*Pr)_6_]_∞_ (M = Rb) derivatives.

Compound	[UNa(O*^i^*Pr)_6_]_∞_ UNa-O*^i^*Pr-2	[UK(O*^i^*Pr)_6_]_∞_ UK-O*^i^*Pr-2	[URb(O*^i^*Pr)_6_]_∞_ URb-O*^i^*Pr-3
U–M	3.587(4)	3.832(1)	4.009(1)
M–U^1^	3.574(4)	3.825(2)	3.781(1)
U–O*_t_*1 [Å]	2.076(8)	2.069(4)	2.077(5)
U–O*_t_*2 [Å]	2.086(6)	2.085(4)	/
U–O*_µ_*_2_1 [Å]	2.156(7)	2.160(3)	2.141(5)
U–O*_µ_*_2_2 [Å]	2.186(7)	2.160(3)	2.161(6)
U–O*_µ_*_2_3 [Å]	2.173(6)	2.154(3)	2.140(5)
U–O*_µ_*_2_4 [Å]	2.160(6)	2.150(4)	2.141(5)
U–O*_µ_*_2_5 [Å]	/	/	2.105(6)
M–O*_µ_*_2_1 [Å]	2.373(8)	2.674(3)	2.894(6)
M–O*_µ_*_2_2 [Å]	2.374(7)	2.665(4)	2.808(6)
M–O*_µ_*_2_3 [Å]	2.369(8)	2.674(4)	2.860(5)
M–O*_µ_*_2_4 [Å]	2.387(7)	2.677(4)	2.848(6)
M–O*_µ_*_2_5 [Å]			3.438(8)
O*_t_*1–U–O*_µ_*_2_3 [°]	169.5(3)	173.9(2)	175.4(2)
O*_µ_*_2_1–U–O*_µ_*_2_2 [°]	71.7(2)	84.8(1)	86.4(2)
O*_µ_*_2_1–M–O*_µ_*_2_2 [°]	72.0(3)	66.1(1)	62.2(2)

## Data Availability

The original contributions presented in this study are included in the article/Supplementary Material. Further inquiries can be directed to the corresponding author.

## Supplementary Materials

The following supporting information can be downloaded at: https://www.mdpi.com/article/10.3390/molecules30112361/s1.

## References

[1] Cotton S. (2024). Lanthanide and Actinide Chemistry.

[2] Kindra D.R., Evans W.J. (2014). Magnetic susceptibility of uranium complexes. Chem. Rev..

[3] Liddle S.T. (2015). The renaissance of non-aqueous uranium chemistry. Angew. Chem. Int. Ed..

[4] Arnold P.L., Love J.B., Patel D. (2009). Pentavalent uranyl complexes. Coord. Chem. Rev..

[5] Gouder T., Eloirdi R., Caciuffo R. (2018). Direct observation of pure pentavalent uranium in U2O5 thin films by high resolution photoemission spectroscopy. Sci. Rep..

[6] Hinatsu Y., Fujino T., Edelstein N. (1992). Magnetic susceptibility of LiUO_3_. J. Solid State Chem..

[7] Liu J.-H., Van den Berghe S., Konstantinović M. (2009). XPS spectra of the U5+ compounds KUO_3_, NaUO_3_ and Ba_2_U2O_7_. J. Solid State Chem..

[8] Graves C.R., Vaughn A.E., Schelter E.J., Scott B.L., Thompson J.D., Morris D.E., Kiplinger J.L. (2008). Probing the chemistry, electronic structure and redox energetics in organometallic pentavalent uranium complexes. Inorg. Chem..

[9] Fox A.R., Bart S.C., Meyer K., Cummins C.C. (2008). Towards uranium catalysts. Nature.

[10] Graves C.R., Kiplinger J.L. (2009). Pentavalent uranium chemistry—Synthetic pursuit of a rare oxidation state. Chem. Commun..

[11] Jones R., Bindschadler E., Karmas G., Yoeman F., Gilman H. (1956). Organic compounds of uranium. III. Uranium (V) ethoxide. J. Am. Chem. Soc..

[12] Jones R., Bindschadler E., Karmas G., Martin G., Thirtle J., Yoeman F., Gilman H. (1956). Organic compounds of uranium. IV. Uranium (V) alkoxides. J. Am. Chem. Soc..

[13] Jones R., Bindschadler E., Blume D., Karmas G., Martin Jr G., Thirtle J., Gilman H. (1956). Organic Compounds of Uranium. V. Derivatives of Uranium (V) Alkoxides. J. Am. Chem. Soc..

[14] Lichtenberg A., Lichtenberg A., Pieper M., Mathur S. (2024). Mononuclear Uranium and Heterobimetallic Actinide (An = Th, U) Alkoxides with Divalent Group 14 Elements (MII = Ge, Sn, Pb). Eur. J. Inorg. Chem..

[15] Cotton F.A., Marler D.O., Schwotzer W. (1984). Dinuclear uranium alkoxides. Preparation and structures of KU2 (OCMe3) 9, U2 (OCMe3) 9, and U2 (OCHMe2) 10, containing [uranium (IV), uranium (IV)], [uranium (IV), uranium (V)], and [uranium (V), uranium (V)], respectively. Inorg. Chem..

[16] Fortier S., Wu G., Hayton T.W. (2008). Synthesis and Characterization of Three Homoleptic Alkoxides of Uranium: [Li (THF)] 2 [UIV (O t Bu) 6],[Li (Et2O)][UV (O t Bu) 6], and UVI (O t Bu) 6. Inorg. Chem..

[17] Mehrotra R., Singh A., Sogani S. (1994). Homo-and hetero-metallic alkoxides of group 1, 2, and 12 metals. Chem. Soc. Rev..

[18] Shah A., Gupta R., Singh A., Mehrotra R. (1991). Synthesis and Characterization of Heterobimetallic Alkoxides of Iron (III) with Alkali Metals and Aluminium. Synth. React. Inorg. Met. Org. Chem..

[19] Mäntymäki M., Ritala M., Leskelä M. (2012). Double metal alkoxides of lithium: Synthesis, structure and applications in materials chemistry. Coord. Chem. Rev..

[20] Veith M., Mathur S., Huch V. (1997). Novel ionogenic heterometal alkoxide derivatives. Chem. Commun..

[21] Schläfer J., Stucky S., Tyrra W., Mathur S. (2013). Heterobi-and trimetallic cerium (IV) tert-butoxides with mono-, di-, and trivalent metals (M= K (I), Ge (II), Sn (II), Pb (II), Al (III), Fe (III)). Inorg. Chem..

[22] Grödler D., Sperling J.M., Rotermund B.M., Scheibe B., Beck N.B., Mathur S., Albrecht-Schönzart T.E. (2023). Neptunium Alkoxide Chemistry: Expanding Alkoxides to the Transuranium Elements. Inorg. Chem..

[23] Veith M., Mathur S., Huch V. (1997). Synthesis and Characterization of New Alkoxotitanates of Yttrium, Barium, and Copper: Single Crystal X-ray Diffraction Structures of Cl2Y {Ti_2_ (OPri) 9},{Ti (OPri) 5} Ba {Ti_2_ (OPri) 9}, and ClCu {Ti_2_ (OPri) 9}. Inorg. Chem..

[24] Veith M., Mathur S., Mathur C. (1998). New perspectives in the tailoring of hetero (bi-and tri-) metallic alkoxide derivatives. Polyhedron.

[25] Mathur S., Veith M., Ruegamer T., Hemmer E., Shen H. (2004). Chemical vapor deposition of MgAl_2_O_4_ thin films using different Mg−Al Alkoxides: Role of precursor chemistry. Chem. Mater..

[26] Meyer F., Hempelmann R., Mathur S., Veith M. (1999). Microemulsion mediated sol-gel synthesis of nano-scaled MAl_2_O_4_ (M= Co, Ni, Cu) spinels from single-source heterobimetallic alkoxide precursors. J. Mater. Chem..

[27] Lee H., Aytuna Z.T., Bhardwaj A., Wilhelm M., Lê K., May B., Mueller D.N., Mathur S. (2023). Controlling Degree of Inversion in MgFe_2_O_4_ Spinel Films Grown in External Magnetic Fields. Adv. Eng. Mater..

[28] Veith M., Mathur S., Kareiva A., Jilavi M., Zimmer M., Huch V. (1999). Low temperature synthesis of nanocrystalline Y3Al5O12 (YAG) and Ce-doped Y3Al5O12via different sol–gel methods. J. Mater. Chem..

[29] Mathur S., Shen H., Rapalaviciute R., Kareiva A., Donia N. (2004). Kinetically controlled synthesis of metastable YAlO_3_ through molecular level design. J. Mater. Chem..

[30] Raauf A., Schlaefer J., Gessner I., Lichtenberg A., Zegke M., Fischer T., Mathur S. (2022). Homo-and heteroleptic lanthanide-iron alkoxides as precursors in materials synthesis. J. Indian Chem. Soc..

[31] Raauf A., Graf D., Gönüllü Y., Sekhar P.K., Frank M., Mathur S. (2021). Synergistic acceptor-donor interplay of Nd_2_Sn_2_O_7_ pyrochlore based sensor in selective detection of hydrogen. J. Electrochem. Soc..

[32] Bohr C., Yu P., Scigaj M., Hegemann C., Fischer T., Coll M., Mathur S. (2020). Atomic scale growth of GdFeO_3_ perovskite thin films. Thin Solid Films.

[33] Raauf A., Leduc J., Frank M., Stadler D., Graf D., Wilhelm M., Grosch M., Mathur S. (2021). Magnetic field-assisted chemical vapor deposition of UO_2_ thin films. Inorg. Chem..

[34] Lichtenberg A., Altuntas K., Fischer T., Karimpour T., Diel S., Aytuna Z., Amadi C.K., Mathur S. (2024). Molecular Transformations for Direct Synthesis of Thorium Dioxide Films. Z. Anorg. Allg. Chem..

[35] Aytuna Z., Bhardwaj A., Wilhelm M., Patrun D., Fischer T., Sharma R., Papakollu K., Kumar R., Mathur S. (2024). Molecular synthesis strategies for binary MO_2_ (M = V, Sn, Ti, Zr, Hf) high-entropy oxides as superior catalysts for enhanced oxygen evolution. J. Eur. Ceram. Soc..

[36] Patrun D., Zhao S., Aytuna Z., Fischer T., Miess M., Hong Z., Mathur S. (2024). Plasma-enhanced SnO_2_-x thin films on copper current collector for safer lithium metal batteries. Nano Energy.

[37] Hirano S.I., Hayashi T., Nosaki K., Kato K. (1989). Preparation of stoichiometric crystalline lithium niobate fibers by sol-gel processing with metal alkoxides. J. Am. Ceram. Soc..

[38] Verma A., Panayanthatta N., Ichangi A., Fischer T., Montes L., Bano E., Mathur S. (2021). Interdependence of piezoelectric coefficient and film thickness in LiTaO_3_ cantilevers. J. Am. Ceram. Soc..

[39] Phulé P.P., Deis T.A., Dindiger D.G. (1991). Low temperature synthesis of ultrafine LiTaO_3_ powders. J. Mater. Res..

[40] Lyu Y., Wu X., Wang K., Feng Z., Cheng T., Liu Y., Wang M., Chen R., Xu L., Zhou J. (2021). An overview on the advances of LiCoO_2_ cathodes for lithium-ion batteries. Adv. Energy Mater..

[41] Billaud J., Clément R.J., Armstrong A.R., Canales-Vázquez J., Rozier P., Grey C.P., Bruce P.G. (2014). β-NaMnO_2_: A high-performance cathode for sodium-ion batteries. J. Am. Chem. Soc..

[42] Bradley D.C. (1989). Metal alkoxides as precursors for electronic and ceramic materials. Chem. Rev..

[43] Mehrotra R. (1988). Synthesis and reactions of metal alkoxides. J. Non-Cryst. Solids.

[44] Graf D., Schläfer J., Garbe S., Klein A., Mathur S. (2017). Interdependence of structure, morphology, and phase transitions in CVD grown VO_2_ and V_2_O_3_ Nanostructures. Chem. Mater..

[45] Nunes G.G., Friedermann G.R., dos Santos J.L.B., Herbst M.H., Vugman N.V., Hitchcock P.B., Leigh G.J., Sá E.L., da Cunha C.J., Soares J.F. (2005). The first thermochromic vanadium (IV) alkoxide system. Inorg. Chem. Commun..

[46] Lichtenberg A., Zegke M., Nichol G.S., Raauf A., Mathur S. (2023). Influence of alkali metal cations on the formation of the heterobimetallic actinide tert-butoxides [AnM 3 (O t Bu) 7] and [AnM2 (O t Bu) 6](An IV = Th, U. MI = Li, Na, K, Rb, Cs). Dalton Trans..

[47] Krieck S., Schüler P., Görls H., Westerhausen M. (2018). Straightforward synthesis of rubidium bis (trimethylsilyl) amide and complexes of the alkali metal bis (trimethylsilyl) amides with weakly coordinating 2, 2, 5, 5-tetramethyltetrahydrofuran. Dalton Trans..

[48] Ojeda-Amador A.I., Martínez-Martínez A.J., Kennedy A.R., O’Hara C.T. (2016). Structural studies of cesium, lithium/cesium, and sodium/cesium bis (trimethylsilyl) amide (HMDS) complexes. Inorg. Chem..

[49] Hampden-Smith M.J., Williams D., Rheingold A. (1990). Synthesis and characterization of alkali-metal titanium alkoxide compounds MTi (O-iso-Pr) 5 (M = Li, Na, K): Single crystal X-ray diffraction structure of [LiTi (O-iso-Pr) 5] 2. Inorg. Chem..

[50] Veith M., Mathur S., Mathur C., Huch V. (1997). Synthesis, reactivity and structures of hafnium-containing homo-and hetero-(bi-and tri-) metallic alkoxides based on edge-and face-sharing bioctahedral alkoxometalate ligands. J. Chem. Soc. Dalton Trans..

[51] Veith M., Rosier R. (1986). Alkoxistannate, II [1] Tri (tert-butoxi) alkalistannate (II): Darstellung und Strukturen/Alkoxistannate, II [1] Tri (tert-butoxi) alkalistannates (II): Synthesis and Structures. Z. Naturforschung B.

[52] Amonoo-Neizer E.H., Shaw R.A., Skovlin D.O., Smith B.C., Rosenthal J.W., Jolly W.L. (1966). Lithium Bis(trimethylsilyl)amide and Tris(trimethylsilyl)amine. Inorganic Syntheses.

[53] Leduc J., Frank M., Jürgensen L., Graf D., Raauf A., Mathur S. (2019). Chemistry of actinide centers in heterogeneous catalytic transformations of small molecules. ACS Catal..

[54] Leduc J., Gönüllü Y., Ruoko T.P., Fischer T., Mayrhofer L., Tkachenko N.V., Dong C.L., Held A., Moseler M., Mathur S. (2019). Electronically coupled uranium and iron oxide heterojunctions as efficient water oxidation catalysts. Adv. Funct. Mater..

[55] Lichtenberg A., Patrun D., Fischer T., Tao J., Halankar K., Hong Z., Mathur S. (2025). From Nuclear Waste to Sustainable Energy: LiUO_3_ as a Stable Anode Material in Lithium-Ion Batteries. ACS Appl. Mater. Interfaces.

