# Supplementary material for: Heterobimetallic Uranium(V)-Alkali Metal Alkoxides: Expanding the Chemistry of f-Block Elements
Source: Molecules. 2025 May 29;30(11):2361. doi: 10.3390/molecules30112361 (PMC12155610; doi:10.3390/molecules30112361)
Supplement: Supplementary file 1 [file molecules-30-02361-s001.zip › molecules-3644820-supplementary.pdf]

Supporting Information

Heterobimetallic Uranium(V) Alkoxides with Monovalent Alkali Metal Cations

*Andreas Lichtenberg, Lidia Inderdühnen, Aida Lichtenberg, Sanjay Mathur**

[1] Institute of Inorganic and Materials Chemistry, University of Cologne; 50939 Cologne (Germany)

*Corresponding Author: sanjay.mathur@uni-koeln.de

Actinides, Uranium, Alkali Metal, Alkoxides, Ternary Oxides.

General Remarks

CCDC deposition numbers 2447401 – 2447408 contain the supplementary crystallographic data for this article. These data can be obtained free of charge via www.ccdc.cam.ac.uk.

S1 Uranium(V)-Alkali Metal(I) *tert*-Butoxides

S1.1 Single Crystal X-Ray Diffraction

Table S1 Compiled crystallographic information of the refined models for the heterobimetallic An(V)M(I) *tert*-butoxide compounds [UNa(O^{t}Bu)$_6$] **UNa-O^{t}Bu**, [UK(O^{t}Bu)$_6$] **UK-O^{t}Bu**, [URb(O^{t}Bu)$_6$] **URb-O^{t}Bu** and [UCs(O^{t}Bu)$_6$] **UCs-O^{t}Bu**.

Compound	UNa-O^{t}Bu	UK-O^{t}Bu	URb-O^{t}Bu	UCs-O^{t}Bu
formula	$UNaO_6C_{24}H_{54}$ · C_6H_6	$UKO_6C_{24}H_{54}$ · C_6H_6	$URbO_6C_{24}H_{54}$ · C_6H_6	$UCsO_6C_{24}H_{54}$
M_r [g mol^{-1}]	777.80	793.91	840.28	809.61
temperature [K]	100.0	100.0	100.0	100.0
crystal system	trigonal	trigonal	trigonal	orthorhombic
space group	*R*3*m*	*R*3*m*	*R*3*m*	*Immm*
a [Å]	15.6450(8)	15.5798(4)	15.5618(5)	11.0099(6)
b [Å]	15.6450(8)	15.5798(4)	15.5618(5)	12.2439(5)
c [Å]	12.4591(9)	12.6997(5)	12.9358(7)	13.9343(8)
α [°]	90	90	90	90
β [°]	90	90	90	90
γ [°]	120	120	120	90
V [Å^3]	2641.0(3)	2669.61(18)	2713.0(2)	1878.40(17)
Z	3	3	3	2
μ (M_0-K_α) [mm^{-1}]	4.657	4.712	5.858	5.301
no. of unique rflns, R_{int}	3089, 0.0513	2774, 0.0629	1502, 0.0407	1547, 0.0406
goodness of fit	1.055	1.062	1.094	1.160
final R indices [I>2σ(I)]: R_1, wR_2	0.0190, 0.0479	0.0209, 0.0436	0.0112, 0.0275	0.0324, 0.0824
R indices (all data): R_1, wR_2	0.0190, 0.0479	0.0209, 0.0436	0.0112, 0.0275	0.0324, 0.0824
largest diff. peak and hole	1.09, -0.90	1.85, -0.89	0.46, -0.73	0.81, -1.86
flack parameter	0.533(14)	0.323(11)	0.405(10)	

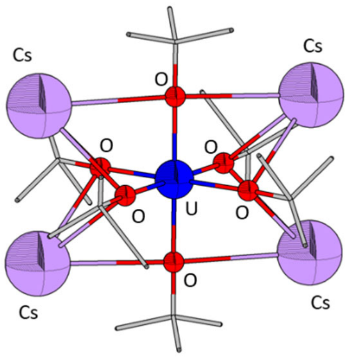

Figure S1 The molecular structure of [UCs(O^{t}Bu)$_6$] (**UCs-O^{t}Bu**) exhibits a fourfold disorder of the Cs center, with each Cs atom having a occupancy of 0.25.

S1.2 Infrared Spectroscopy

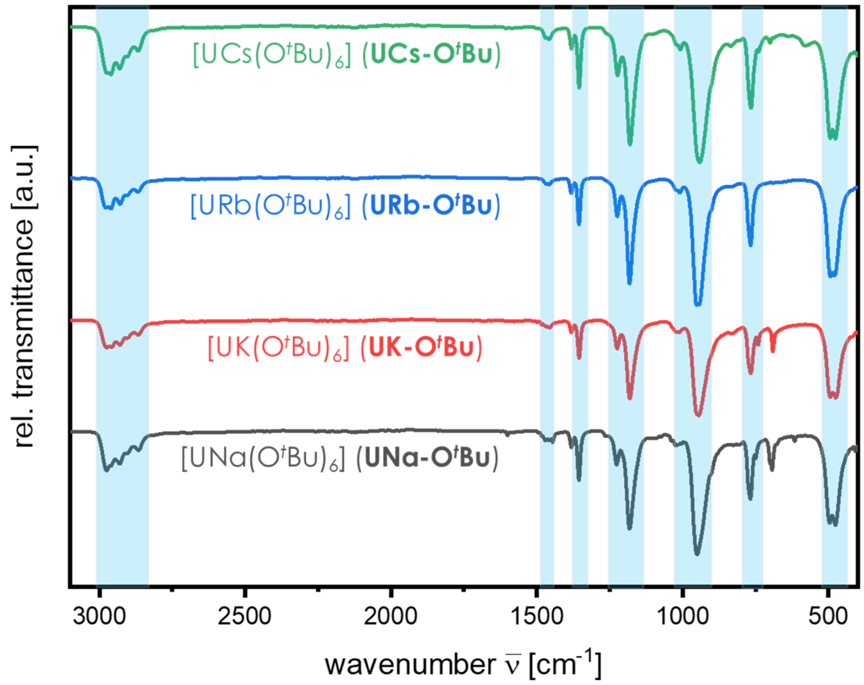

Figure S2 Infrared Spectra of the heterobimetallic $U^{V}M^{I}$ *tert*-butoxide compounds $[UM(O^tBu)_6]$ (UM-O^{t}Bu-type: UNa-O^{t}Bu, UK-O^{t}Bu, URb-O^{t}Bu, UCs-O^{t}Bu).

S2 Uranium(V)-Alkali Metal(I) *iso*-Propoxides

S2.1 Single Crystal X-Ray Diffraction

Table S2 Compiled crystallographic information of the refined models for the heterobimetallic An(V)M(I) *iso*-propoxide compounds $[UM(O^iPr)_6]_n$ (n = 2, M^I = Li (**ULi-O^{i}Pr-1**); n = ∞, M^I = Na (**UNa-O^{i}Pr-2**), K (**UK-O^{i}Pr-2**), Rb (**URb-O^{i}Pr-3**)).

Compound	$[UNa(O^iPr)_6]_n$ **ULi-O^{i}Pr-1**	$[UNa(O^iPr)_6]_\infty$ **UNa-O^{i}Pr-2**	$[UK(O^iPr)_6]_\infty$ **UK-O^{i}Pr-2**	$[URb(O^iPr)_6]_\infty$ **URb-O^{i}Pr-3**
formula	$ULiO_6C_{18}H_{42}$	$UNaO_6C_{18}H_{42}$	$UKO_6C_{18}H_{42}$	$URbO_6C_{18}H_{42}$
M_r [g mol^{-1}]	599.48	615.53	631.64	678.01
temperature [K]	100.0	100.0	100.0	100.0
crystal system	monoclinic	monoclinic	monoclinic	monoclinic
space group	*C*2/*m*	*P*2$_1$/*n*	*P*2$_1$/*n*	*P*2$_1$/*n*
a [Å]	19.7469(8)	11.3826(8)	11.1114(6)	10.8993(4)
b [Å]	9.7065(3)	12.6086(8)	13.3649(8)	14.4497(5)
c [Å]	15.0846(6)	18.7828(13)	18.4548(11)	17.4263(7)
α [°]	90.0	90.0	90.0	90.0
β [°]	115.2520(10)	101.499(2)	102.749(2)	92.836(2)
γ [°]	90.0	90.0	90.0	90.0
V [Å^3]	2615.02(10)	2641.6(3)	2673.0(3)	2741.14(18)
Z	4	4	4	4
μ (M_0-K_α) [mm^{-1}]	6.230	6.185	6.252	7.709
no. of unique rflns, R_{int}	3438, 0.0567	5405, 0.0404	11231, 0.0797	7116, 0.0536
goodness of fit	1.065	1.105	1.019	1.049
final R indices [$I>2\sigma(I)$]: R_1, wR_2	0.0251, 0.0678	0.0621, 0.1685	0.0451, 0.1023	0.0470, 0.1240
R indices (all data): R_1, wR_2	0.0268, 0.0686	0.0847, 0.1839	0.0824, 0.1193	0.0553, 0.1294
largest diff. peak and hole	1.29, -1.00	2.99, -1.83	1.33, -1.05	4.50, -2.59

S2.2 NMR Spectroscopy

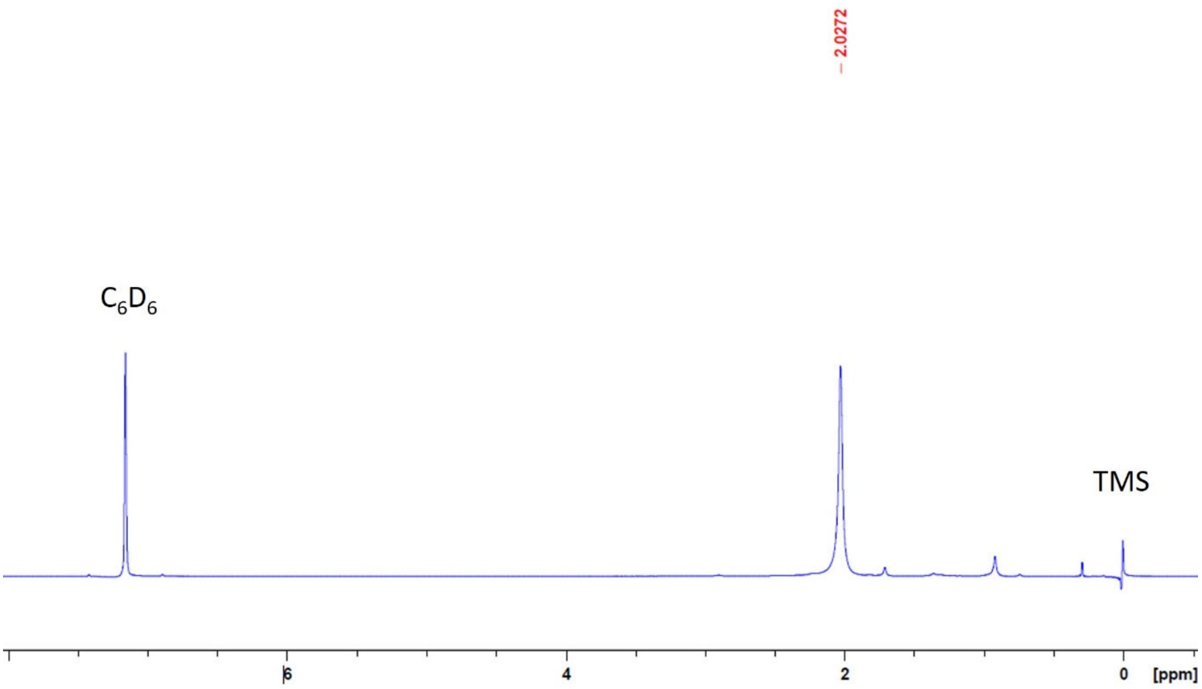

Figure S3 Room temperature ^{1}H NMR spectrum of [UNa(O^{t}Bu)$_6$] (**UNa-O^{t}Bu**) in benzene-d_6 on a 300 MHz *Bruker Avance 300* spectrometer.

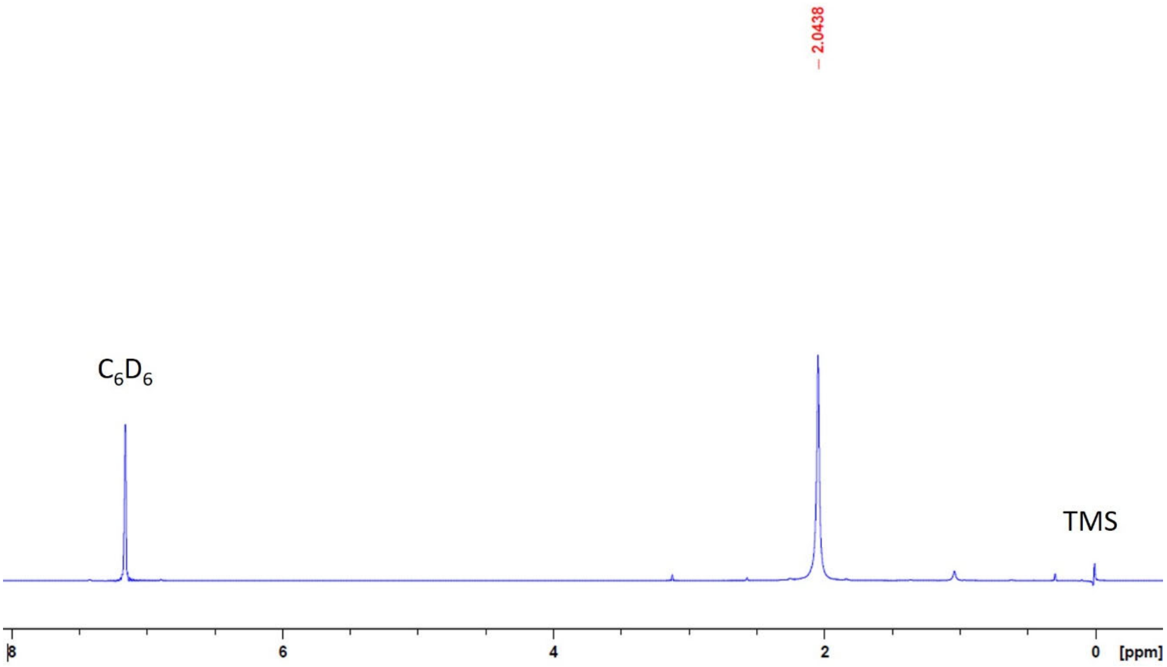

Figure S4 Room temperature ^{1}H NMR spectrum of [UK(O^{t}Bu)$_6$] (**UK-O^{t}Bu**) in benzene-d_6 on a 300 MHz *Bruker Avance 300* spectrometer.

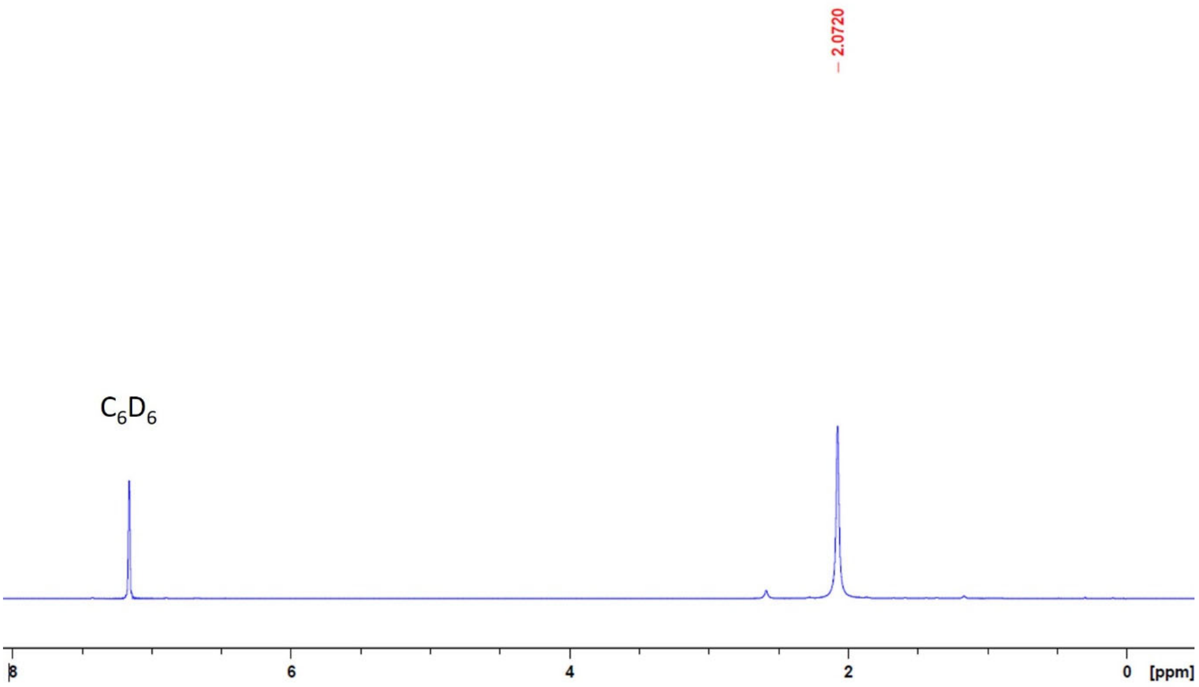

Figure S5 Room temperature ^1H NMR spectrum of $[URb(O^tBu)_6]$ (**URb-O^{t}Bu**) in benzene-d_6 on a 300 MHz *Bruker Avance 300* spectrometer.

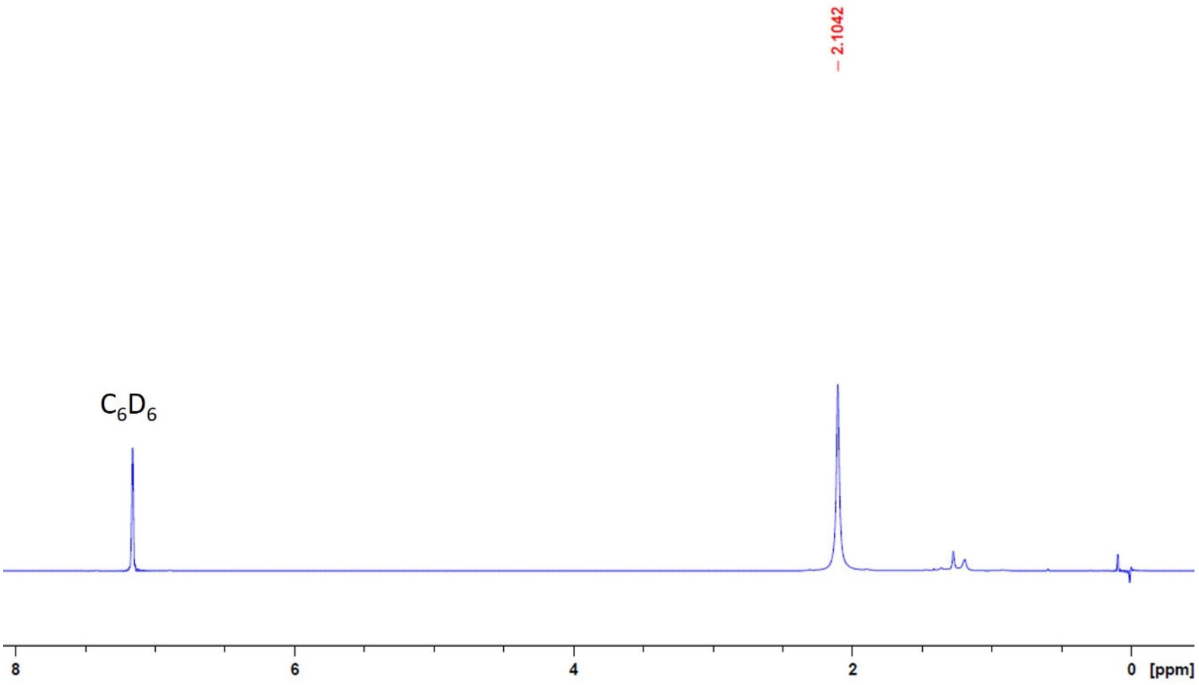

Figure S6 Room temperature ^1H NMR spectrum of $[URb(O^tBu)_6]$ (**UCs-O^{t}Bu**) in benzene-d_6 on a 300 MHz *Bruker Avance 300* spectrometer.

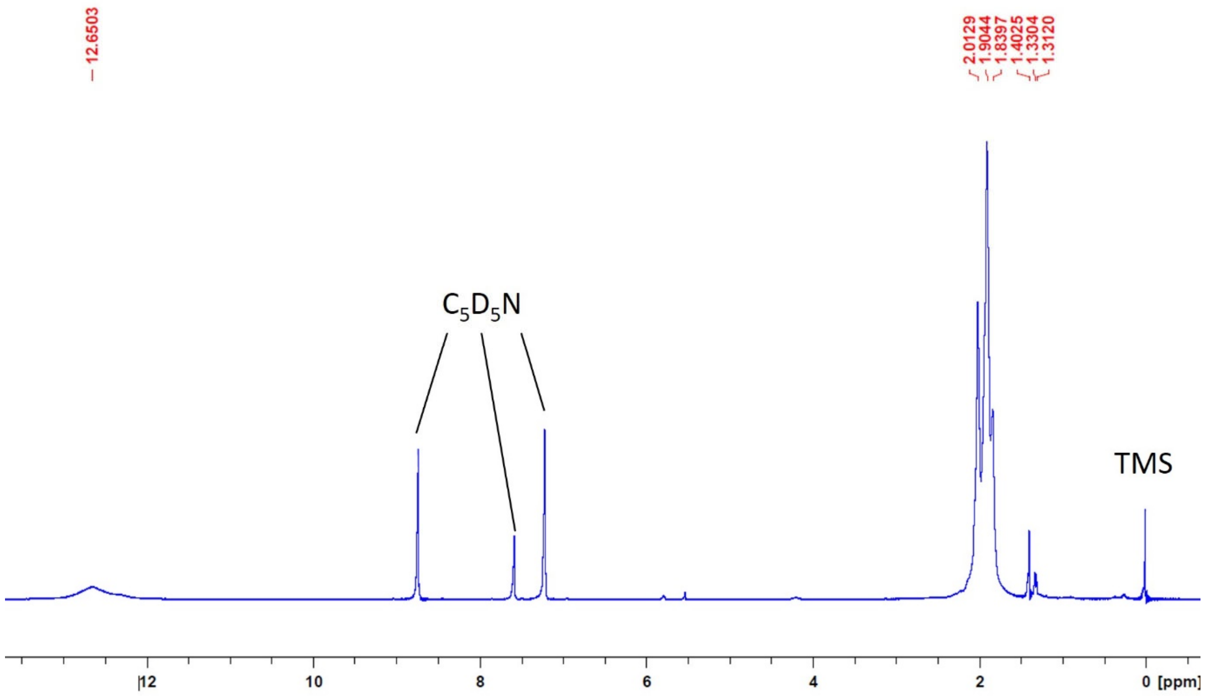

Figure S7 Room temperature ^{1}H NMR spectrum of $[ULi(O^{i}Pr)_6]_2$ (**ULi-O^{i}Pr-1**) in pyridine-d_5 on a 300 MHz *Bruker Avance 300* spectrometer.

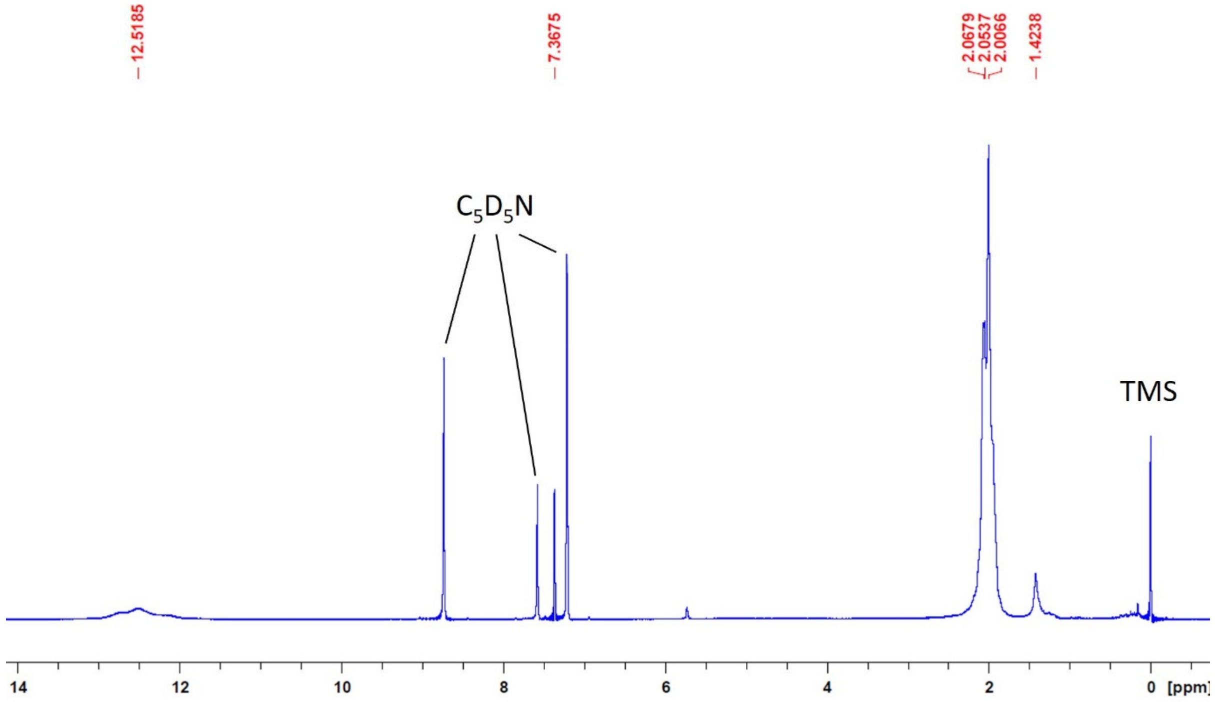

Figure S8 Room temperature ^{1}H NMR spectrum of $[UNa(O^{i}Pr)_6]_\infty$ (**UNa-O^{i}Pr-2**) in pyridine-d_5 on a 300 MHz *Bruker Avance 300* spectrometer.

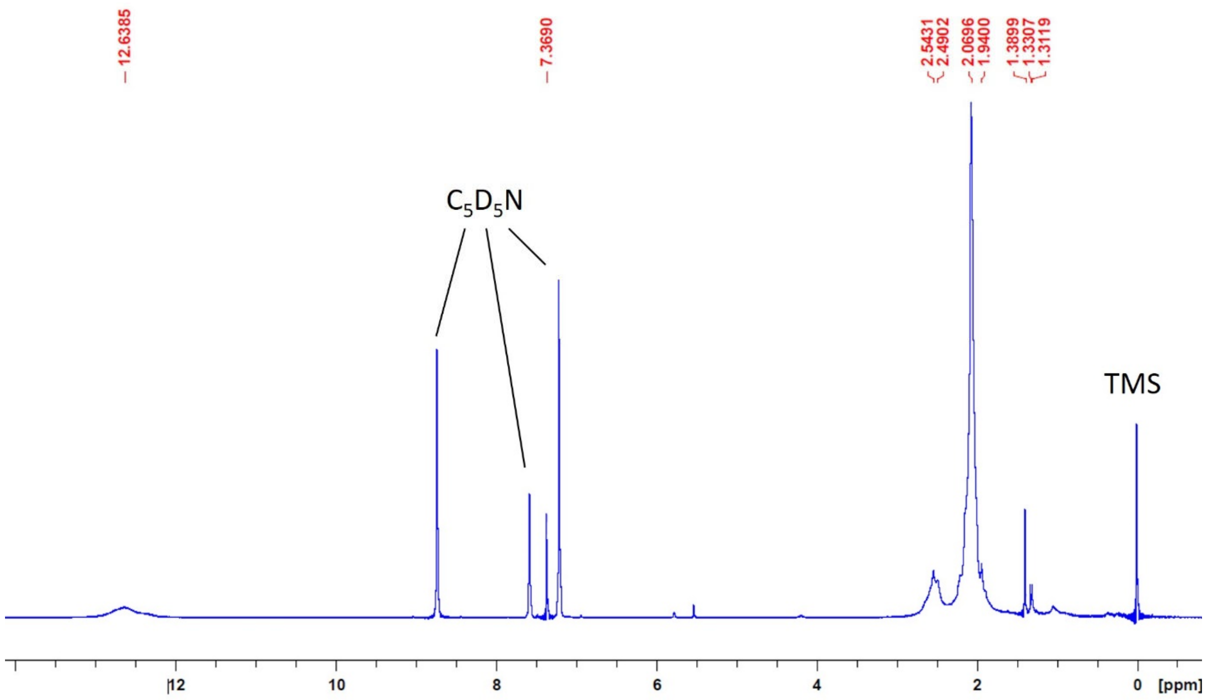

Figure S9 Room temperature ^{1}H NMR spectrum of $[UK(O^{i}Pr)_6]_\infty$ (**UK-O^{i}Pr-2**) in pyridine-d_5 on a 300 MHz *Bruker Avance 300* spectrometer.

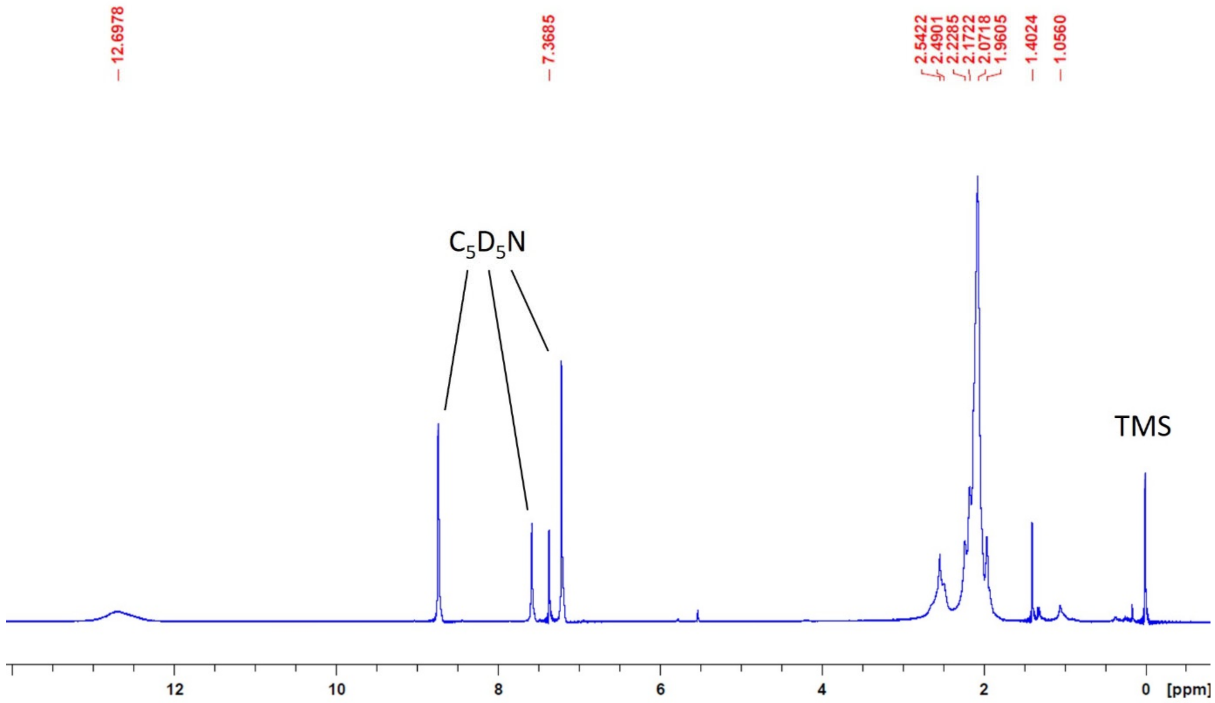

Figure S10 Room temperature ^{1}H NMR spectrum of $[URb(O^{i}Pr)_6]_\infty$ (**URb-O^{i}Pr-3**) in pyridine-d_5 on a 300 MHz *Bruker Avance 300* spectrometer.

S2.3 Infrared Spectroscopy

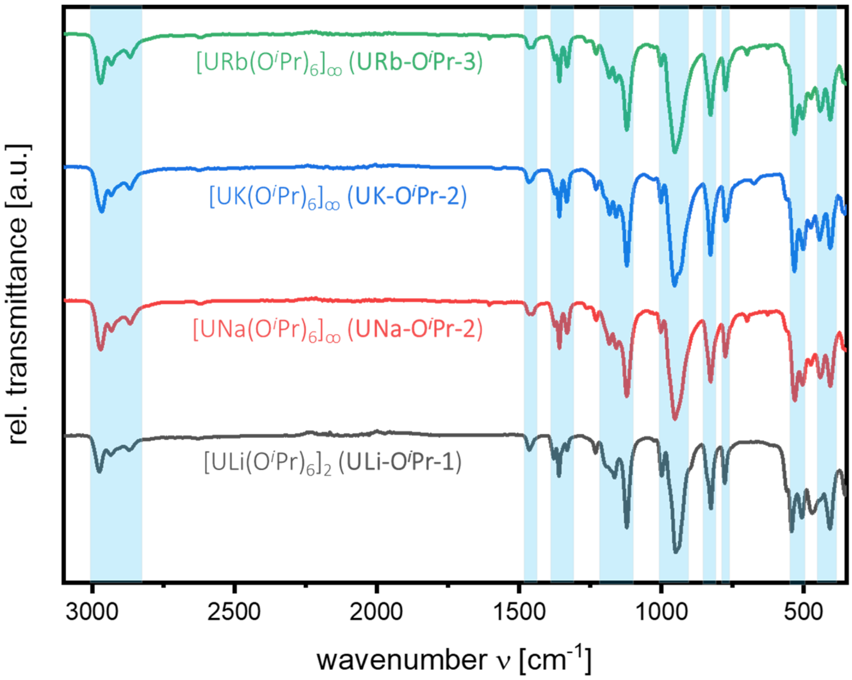

Figure S11 Infrared Spectra of the An^V-M^I containing *iso*-propoxide derivatives [UM(O^{i}Pr)$_6$]$_n$ (n = 2, M^I = Li (**ULi-O^{i}Pr-1**); n = ∞, M^I = Na (**UNa-O^{i}Pr-2**), K (**UK-O^{i}Pr-2**), Rb (**URb-O^{i}Pr-3**)).
